# Expansive microbial metabolic versatility and biodiversity in dynamic Guaymas Basin hydrothermal sediments

**DOI:** 10.1038/s41467-018-07418-0

**Published:** 2018-11-27

**Authors:** Nina Dombrowski, Andreas P. Teske, Brett J. Baker

**Affiliations:** 1 Department of Marine Science, Marine Science Institute, University of Texas Austin, Port Aransas, TX 78373 USA; 2 Department of Marine Sciences, University of North Carolina at Chapel Hill, Chapel Hill, NC 27599 USA

## Abstract

Microbes in Guaymas Basin (Gulf of California) hydrothermal sediments thrive on hydrocarbons and sulfur and experience steep, fluctuating temperature and chemical gradients. The functional capacities of communities inhabiting this dynamic habitat are largely unknown. Here, we reconstructed 551 genomes from hydrothermally influenced, and nearby cold sediments belonging to 56 phyla (40 uncultured). These genomes comprise 22 unique lineages, including five new candidate phyla. In contrast to findings from cold hydrocarbon seeps, hydrothermal-associated communities are more diverse and archaea dominate over bacteria. Genome-based metabolic inferences provide first insights into the ecological niches of these uncultured microbes, including methane cycling in new Crenarchaeota and alkane utilization in ANME-1. These communities are shaped by a high biodiversity, partitioning among nitrogen and sulfur pathways and redundancy in core carbon-processing pathways. The dynamic sediments select for distinctive microbial communities that stand out by expansive biodiversity, and open up new physiological perspectives into hydrothermal ecosystem function.

## Abstract (web summary)

The diversity and function of microbes inhabiting hydrothermal areas is a topic of active interest in marine microbiology. Here, the authors assemble genomes from Guaymas Basin hydrothermal sediments and describe the metabolic roles of the bacterial community, which includes five new bacterial candidate phyla

## Introduction

Microbial communities inhabit every environment and are comprised of a multitude of different phyla, the majority of which are uncultured^1^. Among these environments, marine sediments contain abundant and phylogenetically diverse microbial communities^2–4^. High diversity has been suggested to emerge as a strategy for survival of microbes under fluctuating environmental conditions in nature^5,6^. While single-gene surveys allow us to address the phylogenetic diversity of microbial communities, metagenomic analyses provide a connection between diversity and the functional potential encoded within sedimentary communities.

Guaymas Basin (GB; Gulf of California, Mexico) is a young, active seafloor-spreading center characterized by high water column productivity and fast sedimentation rates, leading to the accumulation of massive layers of organic-rich sediments that cover the hydrothermal spreading center and ridge flanks^7–9^. The emplacement of hot basalt sills into organic-rich sediment transforms buried organic matter into CO_2_, H_2_, low-molecular-weight organic acids, ammonia, and hydrocarbons such as methane, ethane and benzene^8,10,11^. These compounds migrate to the sediment surface with rising vent fluids, where they fuel hydrocarbon-degrading microbial communities^11,12^. Among all hydrothermally generated hydrocarbons, methane has received considerable interest as greenhouse gas shaping global climate^13^. Porewater methane reaches millimolar concentrations while ethane ranges from 40-100 µM. Also present in these sediments are propane, n-butane and pentane, which accumulate at lower concentrations compared to methane. Altogether, hydrocarbons represent lucrative carbon sources for the resident microbial community^11,14–16^. Additionally, hydrothermal circulation and seawater in-mixing provide the upper sediments with electron acceptors, among which sulfate is widely available in millimolar porewater concentrations and rarely depleted within hydrothermal sediment cores^11,14,17^. In-situ microelectrode surveys detect small oxygen peaks within hydrothermal sediments near the mat-covered surface^18,19^. These results are consistent with short-term dynamics of hydrothermal flow within minutes and hours^17^. Additionally, short-term dynamics overlay with longer-term hydrothermal activity changes over months and years^18^.

GB sediments have been shown to host diverse microbial communities with distinct roles in carbon cycling^11,17,20^. In particular, microbial consortia perform the anaerobic oxidation of methane (AOM) in a syntrophic interaction consisting of anaerobic methane-oxidizing archaea (ANME) and bacterial sulfate reducers, typically Deltaproteobacteria, but including other thermophilic bacterial lineages, such as *Candidatus* Desulfofervidus auxilii^21–23^. Anaerobic hydrocarbon degraders include Ca. Syntrophoarchaeum, which oxidizes butane in a syntrophic interaction with Ca. Desulfofervidus auxilii, or the butane- and propane oxidizing isolate BuS5, belonging to the *Desulfosarcina*-*Desulfococcus* cluster^15,16^. Other common archaeal lineages include Marine Benthic Group D and Bathyarchaeota, while bacterial phyla include Proteobacteria (Delta-, Epsilon- and Gammaproteobacteria), Bacteroidetes and Chloroflexi as well as several candidate phyla^11,17,24^. Within the GB hydrothermal area in the southern spreading center, a high degree of microbial community connectivity exists among hydrothermal vent sites and sediments within a few hundred meters^25^. A core microbiome is shared between microbial communities of GB hydrothermal sediments and cold seeps in the Sonora Margin, within a few km distance; this microbiome is thought to be involved in organic matter degradation as well as methane and carbon cycling, suggesting microbial exchanges across neighboring sites that share geochemical characteristics, such as abundant methane concentrations^26^. Previously, we employed metagenomic reconstructions of two GB sedimentary microbial communities, showing the interconnectivity of carbon, sulfur and nitrogen cycling among lineages^20^. However, despite these advances, we still have a limited understanding of the spatial biodiversity and full metabolic potential of microbes inhabiting the basin.

Here we characterize the biodiversity and physiological capabilities of genomes from microbial communities inhabiting GB sediments. The highly localized hydrothermal gradients in surficial GB sediments are ideal to compare adjacent sites with distinct temperature and chemical regimes^18,27^. We selected samples from methane- and sulfate-rich hydrothermal sediments covering a wide thermal range, and contrasted them with cold, non-hydrothermal sediments, as well as with hot, oil-rich sediments. We hypothesize that microbial assemblages from hydrothermal sediments are phylogenetically distinct from those in the surrounding region and host a greater metabolic diversity. Therefore, we sequenced a total of ~4 billion genomic reads from eleven samples (two of which were from cool, background sediments) from GB. Altogether, these data add 22 branches to the tree of life and enabled to us determine the genetic repertoire and metabolic versatility of these extreme hydrothermal communities.

## Results

### Phylogenetic diversity in Guaymas Basin sediments

**Fig. 1:**
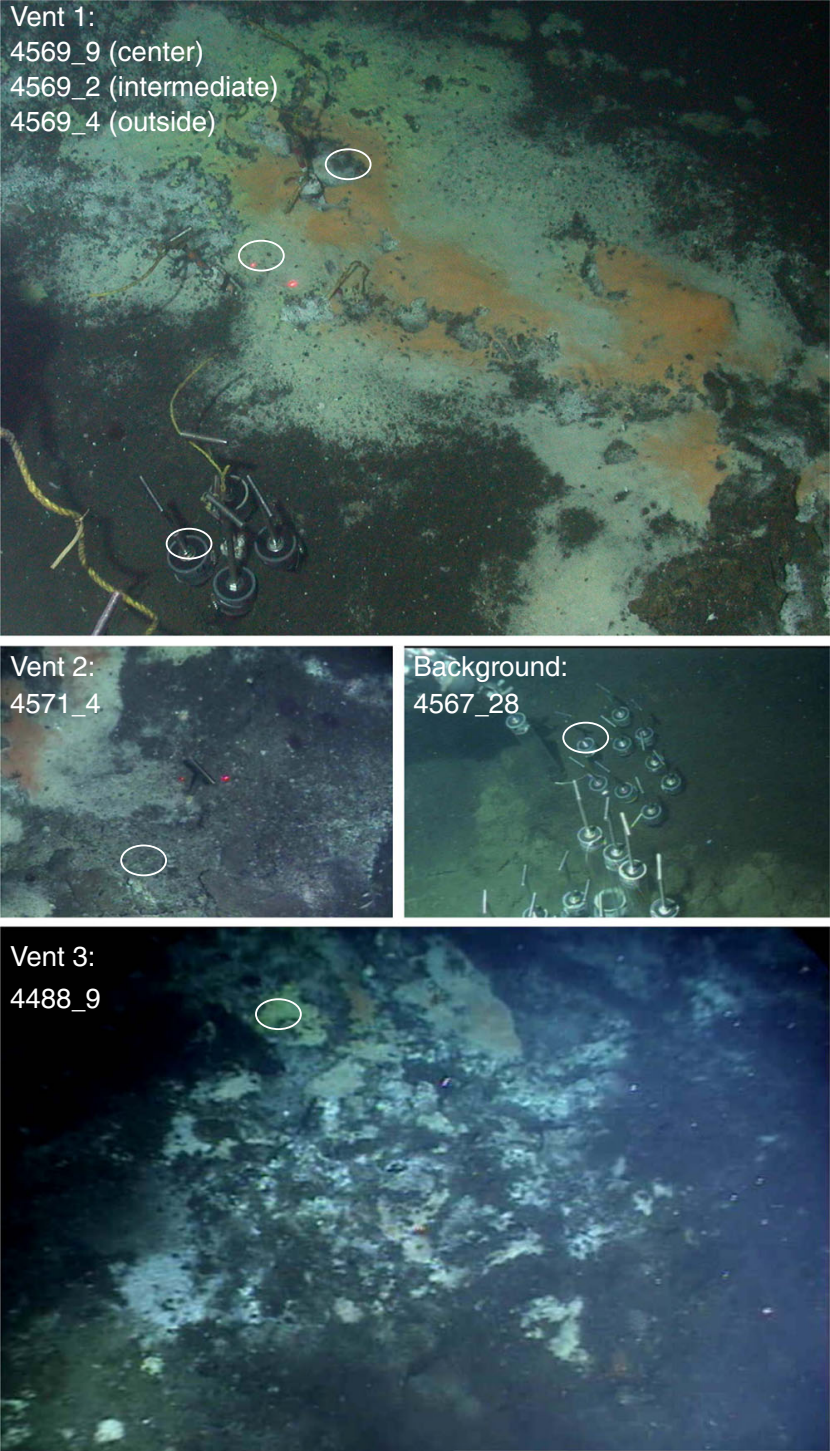
Overview of sampling sites. In-situ photos of the three hydrothermal sampling sites (Vent1, Vent2, Vent3) and the non-hydrothermal background sediment, including Alvin dive and core number for the sediment cores that were used for DNA extraction and metagenomic analysis. White circle: spots where sediment cores were retrieved by push coring. For Vent1, three sediment cores were taken inside the yellow mat (4569_9), further outside in a white mat area (4569_2) and outside of the mat area (4569_4); next to each core a thermal logging probe was inserted into the sediment. At Vent2 and Vent3, one core each (4571_4 and 4488_9) was sampled. Metadata for all samples are summarized in Supplementary Data 1

To examine the biodiversity of microbial communities inhabiting GB sediments, we sampled and sequenced eleven sediments covering different sampling locations, depths (0–24 cm), temperatures (3–60 °C) and geochemical regimes (Fig. 1, Supplementary Data 1, 2). Background sediments, represented by core 4567_28, are not influenced by hydrothermal activity (temperature ~3 °C) and occur interlaced with hydrothermal hot spots within the spreading center^18^. All other samples are characterized by steep thermal gradients, reflected by *in-situ* temperatures ranging from 4 °C to 60 °C. Dense mats of filamentous *Gammaproteobacteria* (family *Beggiatoaceae*) covered hydrothermal sediments from dive 4569, with an orange mat dominating core 4569_9 and a white mat at the adjacent core 4569_2. Core 4569_4 was collected from the periphery of this hydrothermal hotspot and did not contain visible mats (Fig. 1). Porewater methane, sulfate, dissolved inorganic carbon (DIC) and sulfide co-occurred throughout these cores (Supplementary Information), consistent with hydrothermal circulation and inmixing of seawater-derived electron acceptors. Cores 4571_4 and 4488_9 represent hot and oily sediments with yellow-white sulfur precipitates on the surface (Fig. 1). Among the hydrothermal cores, 4488_9 stands out by steep thermal gradients (~150 °C at 30 cm depth), high sample temperature (~60 °C), sulfate depletion at shallow depths, and accumulation of non-methane hydrocarbons (Supplementary Figure 1, Supplementary Data 1). After sequence assembly, we reconstructed 551 draft genomes via tetranucleotide and coverage binning. These metagenome-assembled genomes (MAGs), simplified as ‘genome’ throughout the manuscript, represent medium-quality MAGs and were > 50% complete and < 10% contaminated (301 genomes > 70% and 61 genomes > 90% complete; Supplementary Data 2, 3)^28^.

**Fig. 2:**
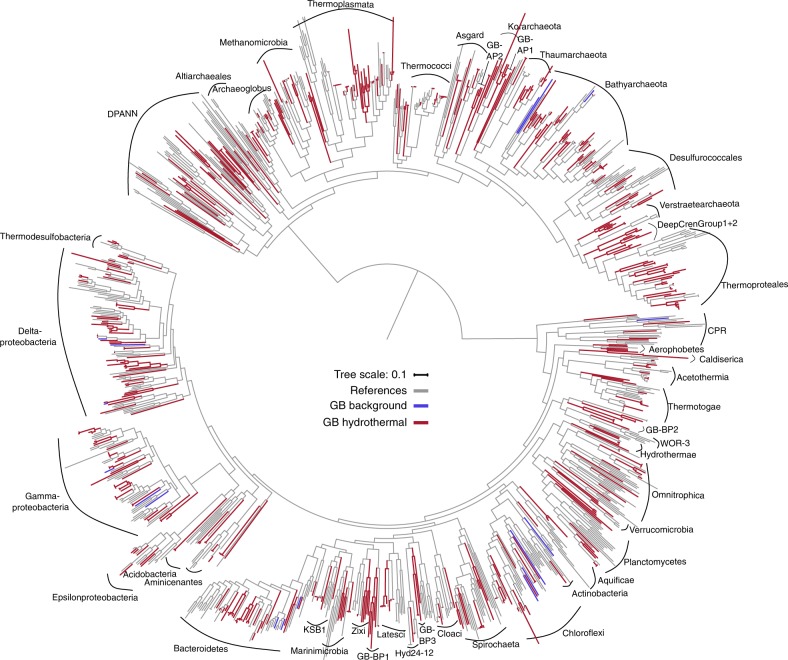
Maximum likelihood phylogenetic tree of GB genomes based on 37 concatenated protein-coding genes. Grey: Reference Genomes. Blue: Genomes assembled from cold background sediments. Red: Genomes recovered from hot, hydrothermal sediments. The full tree can be found in Supplementary Figure 2 and the tree file is available in Supplementary Data 5

Each genome was classified by constructing a phylogenetic tree using 37 single-copy, protein-coding marker genes (Supplementary Data 4)^29^. Overall, the 551 genomes (247 archaea and 304 bacteria) represented 16 cultured and 40 uncultured, candidate phyla that comprise a substantial number of new microbial lineages, many of which branch basal to those previously described (Fig. 2, Supplementary Figure 2, Supplementary Data 5). GB genomes form 22 new lineages on the tree of life based on a phylogenetic distance analysis (collapsing branches at an average branch length distance < 0.6). Among those lineages, we discovered five new candidate phyla designated GB-AP1,2 and GB-BP1-3 for archaeal and bacterial phyla, respectively. The placement of these five phyla was confirmed by comparing the average amino acid identity (AAI) of genomes within a phylum to genomes of all other phyla (Supplementary Data 6). Within each new phylum, GB-AP1,2 shared an AAI of ~44 and ~96% and GB-BP1-3 of ~54, ~72 and ~60%, respectively, and were more similar to themselves then to other genomes (~43% AAI summarized across all genomes). While the genomes of GB-AP1 shared a low AAI, we did not detect any lineage with a closer similarity. Two 16S rRNA gene sequences recovered from GB-BP1 clustered with the uncultivated lineage MAT-CR-M4-B07^30^, which was previously detected in the Kazan mud volcano or Guerrero Negro hypersaline mats (Supplementary Figure 3). In total, we defined 24 archaeal and 37 bacterial groups (or ‘clusters’) for closer analysis (see Methods section, Supplementary Data 3 and Fig. 2). Archaeal genomes were represented by Bathyarchaeota (*n* = 41), Thermoproteales (*n* = 40) and Thermoplasmata (*n* = 36), and bacteria belonged to Deltaproteobacteria (*n* = 56), Gammaproteobacteria (*n* = 39) and Bacteroidetes (*n* = 27; Supplementary Data 3). Additionally, we detected several candidate lineages, including Asgard archaea (*n* = 9), Verstraetearchaeota (*n* = 7) and the bacterial CPR superphylum (*n* = 6). Overall, more genomes were recovered from hydrothermal (average of ~60 genomes per sample) than from background sediments (average ~9 genomes per sample; Supplementary Data 3). We detected only one archaeal (Bathyarchaeota) and 7 bacterial lineages (Chloroflexi, Deltaproteobacteria, Gammaproteobacteria) in the background compared to 22 archaeal and 31 bacterial clusters in the hydrothermal samples, suggesting a greater biodiversity in the more extreme environment.

### The effect of environmental parameters on community assembly

To better understand the factors that drive community assembly, we investigated the occurrence of major phylogenetic clusters across sites. First, we confirmed that the genomes accurately reflected the community as a whole based on the abundance of ribosomal protein S3 across sites (Supplementary Figure 4). Next, we used the genomes to estimate the occurrence of different phylogenetic groups across all samples (Supplementary Figure 5, Supplementary Data 7).  Several bacterial lineages, such as Planctomycetes or Deltaproteobacteria, were more frequently detected in background sediments than in hydrothermal sediments. In contrast, archaea were increasingly detected within the deeper, hotter hydrothermal samples, but not in cool surface sediments on the periphery of hydrothermal hot spots. Dominant lineages in the hot samples were Thaumarchaeota and Archaeoglobales as well as Acetothermia, and Omnitrophica. Two genotypes dominated hot sediments: B48_G6 (Methanosarcinales, ANME-1) and B16_G6 (Thermodesulfobacteria, ~88% AAI to Ca. Desulfofervidus auxilii) (Supplementary Data 3, Supplementary Data 7). While the hydrothermal sediments had an overall similar distribution of taxa across depth profiles, the oily sediment from 4488_9 harbored only few abundant taxa, including Thermoplasmata, Aerophobetes and Thermotoga (Supplementary Figures 4, 5). Core 4488_9 differs from other hydrothermal samples in its high hydrocarbon content, quick downcore depletion of sulfate, and steep thermal gradients (Supplementary Data 1, Supplementary Methods). In combination these factors appear to reduce the microbial diversity, especially of the archaeal community. Altogether, the hydrothermal activity gives rise to a unique community that shows a marked enrichment in archaea that can represent up to 50% of recovered genomes (Supplementary Data 7). This enrichment appears to be largely driven by the rich substrate availability, by hydrothermal circulation and by inmixing of the electron acceptor sulfate (Supplementary Methods). However, a greater sampling size would be needed to disentangle the relative contribution of individual factors on community assembly such as temperature, methane or hydrocarbon availability.

### Carbon cycling

**Fig. 3:**
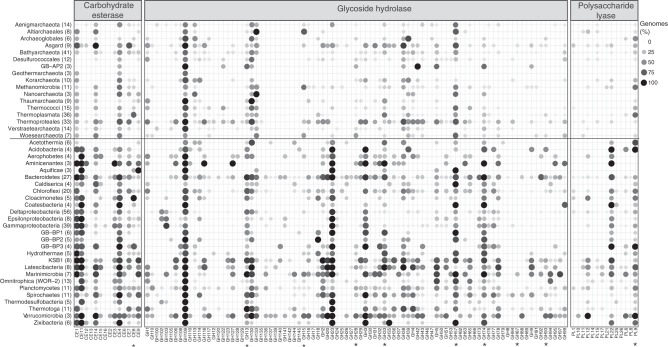
Number of carbohydrate-active enzymes (CAZymes) encoded in GB genomes. Percentage of carbohydrate esterases (CE), glycoside hydrolases (GH) and polysaccharide lyases (PL) encoded in GB genomes summarized for each phylogenetic cluster. Brackets: Total number of genomes encoded in each phylogenetic cluster. Asterisk: CAZyme with potential secretion signal (see also Supplementary Data 8)

Given that these genomes yielded such a large number of unique microbial lineages, we inferred their potential physiological capabilities by assigning metabolic functions to proteins in each individual genome. First, we investigated the ability of the community to degrade and metabolize complex carbohydrates and peptides deposited in sediments by searching genomes for the presence of carbohydrate-active enzymes (CAZYmes), peptidases and pathways for carbon metabolism. In total, we detected ~30,000 and ~11,000 potential CAZYmes and peptidases, respectively (Fig. 3, Supplementary Figure 6, Supplementary Data 8, 9). Generally, bacteria encoded for a broader repertoire of CAZYmes compared to archaea; for example GH13 (α-amylase), GH23 (lytic transglycosylase) or GH74 (xyloglucanase) were more common in bacteria (Fig. 3, Supplementary Data 8). Most CAZymes were assigned to Thermoproteales (*n* = 20) and Asgard archaea (*n* = 16) as well as Verrucomicrobia (*n* = 38) and Bacteroidetes (*n* = 30). Peptidases were more equally distributed across both domains and abundant in Asgard archaea (*n* = 34) and Thermococci (*n* = 24) as well as Aminicenantes (*n* = 54) and Acidobacteria (*n* = 51; Supplementary Figure 6, Supplementary Data 9). Approximately 2–3% of CAZYmes and peptidases are potentially secreted, suggesting that complex substrates are degraded outside of the cell and later taken up for degradation. Potentially secreted enzymes include CE8 (pectin methylesterase), and GH13 (α-amylase) as well as M28 (aminopeptidases) and S08 (subtilisin-like peptidases). A subset of CAZymes, such as GH23, may be involved in cell wall maintenance; however, the presence of sugar and peptide transporters as well as downstream metabolic pathways in most genomes suggest that other CAZymes might be involved in energy metabolism (see below).

**Fig. 4:**
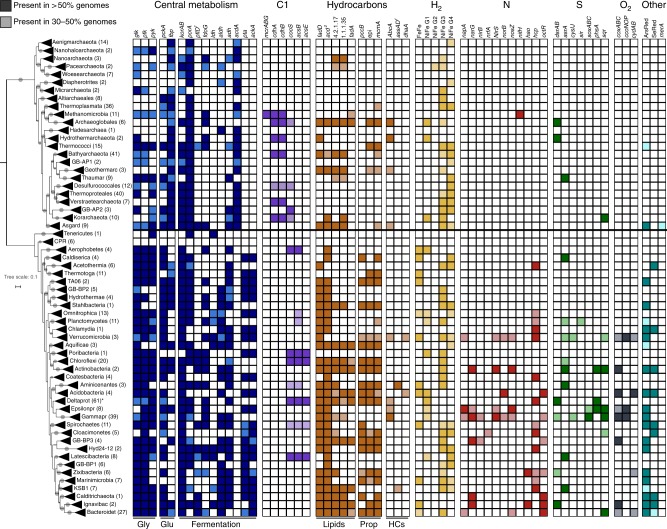
Core metabolic genes detected across phylogenetic clusters inhabiting GB sediments. Presence of core metabolic genes involved in carbon metabolism, hydrocarbon (HC) degradation and respiration. Shaded colors: Gene present in 30–50% of genomes/phylogenetic cluster. Solid colors: Gene present in 50–100% of genomes/cluster. C1 C1- compound metabolism, H2 hydrogen metabolism, N nitrogen metabolism, S sulfur metabolism, O_2_ oxygen metabolism, ArsRed arsenate reductase, SeRed selenate reductase, Gly glycolysis, Glu gluconeogenesis, Prop propane. Number in brackets: number of genomes belonging to individual phylogenetic clusters. Grey circle: Bootstrap support > 70%. Asterisk: Deltaproteobacteria includes genomes from both Deltaproteobacteria and Thermodesulfobacteria. ^1^*pflD* and *assA* are often difficult to discriminate from other glycyl radical enzymes, therefore, an additional phylogenetic analysis can be found in Supplementary Figure 8. ^2^Phylogenetical analyses of substrate specificity of *acd* genes can be found in Supplementary Figure 7. A complete list of metabolic genes can be found in Supplementary Data 10

Common pathways for the degradation of substrates produced by the activity of CAZymes and peptidases include glycolysis (glucokinase (*glk*), phosphofructokinase (*pfk*), pyruvate kinase (*pyk*)), gluconeogenesis (fructose-1,6-bisphosphatase (*fbp*), phosphoenolpyruvate carboxykinase (*pckA*)) and fermentation (Fig. 4 and Supplementary Data 10). In several cases, archaeal genomes encoded for more key genes of gluconeogenesis compared to glycolysis, which could imply that some archaea prefer peptides as an energy source; this finding is consistent with the occurrence of a high number of peptidases in their genomes (Supplementary Figure 6). Compared to archaea, bacteria contained a greater metabolic repertoire and might use both glycolysis and gluconeogenesis. Most genomes encoded for the potential to metabolize pyruvate produced during glycolysis to acetyl-CoA and further into fermentation pathways, producing formate, ethanol or acetate (Fig. 4). GB archaea were mainly capable of acetate formation using the ADP-forming acetyl-CoA synthetase (*acdA*), while bacteria encoded for phosphate acetyltransferase (*pta*) and acetate kinase (*ackA*) for acetate production; formate C-acetyltransferase (*pflD*) and formate dehydrogenase (*fdoG*) for formate production; and aldehyde dehydrogenase (*aldh*) and alcohol dehydrogenase (*adh*) for ethanol production.

Not only is the GB microbiome able to process the deposited organic carbon pool by fermentation, but we also detected pathways for carbon fixation. The most common route of carbon fixation was the Wood-Ljungdahl pathway in both archaea and bacteria, while the Calvin-Benson-Bassham (CBB) and rTCA cycles were restricted mostly to Proteobacteria (Fig. 4, Supplementary Data 10). Although the Group III Ribulose-1,5-bisphosphate carboxylase-oxygenase (Rubisco, key marker gene of the CBB cycle) was detected in most archaea, this subgroup is implied in a nucleotide salvage pathway and not necessarily used for carbon fixation (Supplementary Data 10)^31^. A Group I/II Rubisco, feeding CO_2_ into the CBB cycle, was only detected in some Gammaproteobacteria (orders Chromatiales and Thiotrichales). Additionally, marker genes for the rTCA cycle, including ATP-citrate-lyase (*aclAB*), pyruvate ferredoxin oxidoreductase (*porABCD*) and 2-oxoacid ferredoxin oxidoreductase (*oorABCD*), were mainly detected in Epsilonproteobacteria (order Campylobacterales; Supplementary Data 10). While several genes of the 3-hydroxypropionate or related cycles were present in a subset of genomes, a full pathway appeared to be absent (Supplementary Data 10). Conversely, the Wood-Ljungdahl pathway was present in several clusters, including Archaeoglobales and Methanosarcinales as well as Chloroflexi and Deltaproteobacteria (Fig. 4, Supplementary Data 10). Interestingly, we also detected genes from this pathway in candidate phyla, including Hydrothermarchaeota and Latescibacteria, which might either oxidize acetate or perform acetogenesis.

### Alkyl-coenzyme M reductase linked hydrocarbon cycling

**Fig. 5:**
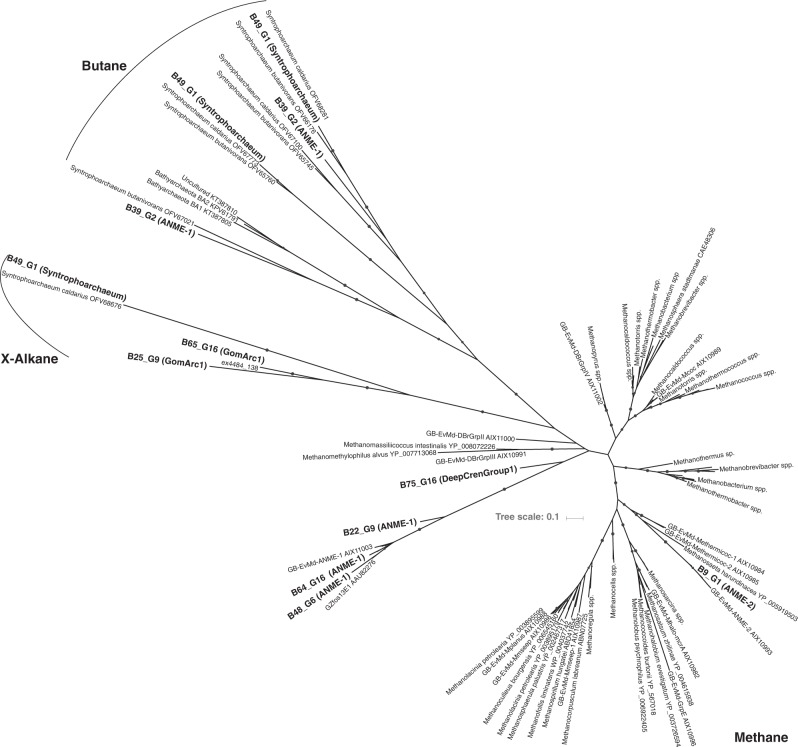
Maximum likelihood phylogenetic tree of the methyl-Coenzyme M reductase (McrA) protein detected in GB genomes. Bold labels: McrA detected in GB genomes (see also Supplementary Data 10). Black circle: Bootstrap support ≥ 70 (number of bootstraps determined using the extended majority-rule consensus tree criterion). RaxML was run as raxmlHPC-PTHREADS-AVX -f a -m PROTGAMMAAUTO -N autoMRE. The tree file is available in Supplementary Data 11

We detected the methyl-Coenzyme M reductase (*mcrA*), a key enzyme for methanogenesis and AOM, in Syntrophoarchaea, Methanomicrobia, and a deep-branching Thermoproteales lineage (designated DeepCrenGroup1; Fig. 4, Supplementary Data 3). To our knowledge this is the first report of *mcrABG* genes in the Crenarchaeota. The only bacteria able to utilize methane encoded for the particulate methane monooxygenase (*pmoA*), which was restricted to Gammaproteobacteria (orders Cellvibrionales and Methylococcales; Supplementary Data 10). A closer phylogenetic analysis of McrA might even suggest a broader substrate usage potentially not restricted to methane (Fig. 5, Supplementary Data 11). McrA from most ANME-1, ANME-2c and DeepCrenGroup1 clustered with known methane oxidizers, while the McrA from one Syntrophoarchaeum (B49_G1) clustered with butane-oxidizers (Fig. 5**)**. McrA from GoM-Arc1 branched between those two clusters, which is consistent with earlier work that suggested that GoM-Arc1 might utilize a different alkane, perhaps ethane, which can reach relatively high concentrations of 40-100 µM in GB^11,20^. However, further experimental evidence, preferably from enrichment cultures, is needed to confirm the substrate usage of these McrA proteins.

Surprisingly, ANME-1 bin B39_G2 contains two McrA proteins (on two different contigs, both mate-paired to other contigs from that bin) that are phylogenetically related to those from Ca. Syntrophoarchaeum spp. (Fig. 5). Similarly to Ca. Syntrophoarchaeum spp. B39_G2 contains genes with homology to those that encode for the butyryl-CoA oxidation pathway, such as acyl-CoA dehydrogenase and enoyl-CoA dehydratase (Supplementary Data 10, Supplementary Figure 7). This pathway appears to be involved in butane oxidation in Ca. Syntrophoarchaeum butanivorans^16^, making this the first example of an ANME-1 archaeon potentially able to use short-chain alkanes. The detection of these unique methyl coenzyme-M reductase genes and pathways suggests that ANME-1 archaea are not limited to methane utilization and potentially able to oxidize alkanes anaerobically.

### Lipid and hydrocarbon utilization

Pathways for lipid degradation were widespread in bacteria and less common in archaea, where they were mainly detected in Archaeoglobales, Bathyarchaeota and Geothermarchaeota (Fig. 4, Supplementary Data 10). The acyl-CoA dehydrogenase (*acd*) represents a key gene catalyzing the first step in beta-oxidation and accommodates a broad substrate range^32,33^. GB ACDs fell alongside described glutaryl-CoA dehydrogenases, small/medium- and long-chain acyl-CoA dehydrogenases, potential butyryl-CoA dehydrogenases and isovaleryl-CoA dehydrogenases (Supplementary Figure 7, Supplementary Data 12). Only ~50% of archaeal lineages encoded for *acd*, which was found scattered across taxa, for example only ~30% of Verstraetearchaeota encoded for *acd*. This gene was common in Archaeoglobales, Asgard archaea and Geothermarchaeota, all of which encoded for other beta-oxidation genes, such as enoyl-CoA hydratase (EC 4.2.1.17) or 3-hydroxyacyl-CoA dehydrogenase (EC 1.1.1.35; Fig. 4, Supplementary Data 10). In contrast, 33 out of 37 bacterial lineages encoded for *acd*. However, only a subset of those lineages - including Aquificae, Chloroflexi or Deltaproteobacteria - encoded for further beta-oxidation genes. In these cases, enzymes, such as the glutaryl-CoA dehydrogenase, might be involved in amino acid catabolism or in benzoyl-CoA degradation^32,34^.

Hydrocarbons are another abundant source for energy and biomass generation in GB. While we did not detect genes for aerobic hydrocarbon degradation, we found indications that GB genomes might anaerobically degrade hydrocarbons using glycyl radical enzymes (GREs, Supplementary Figure 8, Supplementary Data 13). GREs use a radical-based chemistry to carry out challenging metabolic reactions under anaerobic conditions and are involved in a multitude of pathways, such as fermentation, DNA synthesis or hydrocarbon degradation^35,36^. Compared to ACDs, GREs had a sparser distribution and were found in only 6 out of 24 archaeal and 21 out of 37 bacterial lineages. GREs were common in Deltaproteobacteria (*n* = 32), Bacteroidetes (*n* = 23) or Asgard archaea (*n* = 15). Several GREs encoded for enzymes involved in anaerobic hydrocarbon degradation, such as benzylsuccinate synthase (*bssA*) in Deltaproteobacteria (B38_G6, B7_G9), alkylsuccinate synthase (*assA*) in Deltaproteobacteria (B2_G1, B111_G9) or hydroxyphenylacetate decarboxylase in Bathyarchaeota (B26_G17) and Chloroflexi (B43_G15). Some GREs grouped neither with the previously mentioned enzymes nor with the pyruvate formate lyase or other characterized GREs^35^, suggesting that those might utilize different substrates, such as carbohydrates or peptides.

### Respiratory processes

Next, we investigated the GB microbial communities for their involvement in respiratory processes. Overall, more bacterial and archaeal genomes contained genes that encode anaerobic rather than aerobic respiratory pathways, consistent with rapidly depleted oxygen levels within the first few millimeters of the sediment (Fig. 4, Supplementary Data 10)^18^. Cytochrome c oxidases occurred in ~10% of genomes, but were mainly limited to Bacteroidetes, Epsilon-/and Gammaproteobacteria, and Verrucomicrobia. Conversely, genes for hydrogen, nitrogen, sulfur and potentially arsenate and selenate cycling were more widespread. We detected [FeFe]-hydrogenases in ~10% of genomes and these mostly belonged to Group A, which can be involved in fermentative hydrogen evolution^37^. Approximately 70% of genomes encoded for [NiFe]-hydrogenases belonging to Group 1 (a–e and h; membrane-bound hydrogen-uptake hydrogenases involved in hydrogenotrophic respiration), Group 3 (a–d; cytosolic bidirectional hydrogenases) and Group 4 (b,d,e and g; membrane-bound, hydrogen-evolving hydrogenases; Supplementary Data 10)^37^. The most common [NiFe]-hydrogenase was found in ~25% of genomes, and belongs to Group 3b that is involved in NADPH oxidation coupled to hydrogen evolution.

Genes involved in the nitrogen and sulfur cycle were mostly restricted to bacteria, whereas archaeal nitrogen cycling genes were limited to *nifH* in Methanomicrobia (Fig. 4, Supplementary Data 10). Genes for dissimilatory nitrate reduction to ammonium (DNRA) (*narGH*/*napAB* and *nirBD*/*nrfAH*) were present in few Bacteroidetes (i.e. B27_G6, B58_G6), Epsilonproteobacteria (B6_G4, B37_G6) and several Gammaproteobacteria (Methylococcaceae, Thiotrichales). More commonly, we detected DNRA genes distributed separately over several genomes. A complete denitrification pathway (*napA*/*narGH*, *nirK*/*nirS*, *norBC*, *nosZ*) was present in a few genomes, including one Bacteroidetes (B2_G4), some Epsilonproteobacteria (i.e. B135_G9) and several Gammaproteobacteria genomes (Halieaceae, Thiotrichales); individual denitrification genes were found scattered across different taxonomic lineages. Genes involved in anaerobic ammonium oxidation (anammox) were not found, consistent with low nitrate and nitrite concentrations in GB sediments^38^. Genes for the dissimilatory reduction of sulfate to sulfide (*sat*, *aprAB* and *dsrAB*) were found in few archaea (i.e. Archaeoglobales) and several bacteria including Deltaproteobacteria, Gammaproteobacteria and Zixibacteria. The sulfur-oxidation (SOX) system (*soxAX*, *soxYZ*, *soxB*, *soxCD*) showed a restricted phylogenetic distribution and was only located in Epsilonproteobacteria and Gammaproteobacteria. While on average ~10% of all genomes contained genes for sulfur and nitrogen cycling, complete pathways for these processes were present in only few genomes.

### Redundancy and interconnectivity among GB microbes

**Fig. 6:**
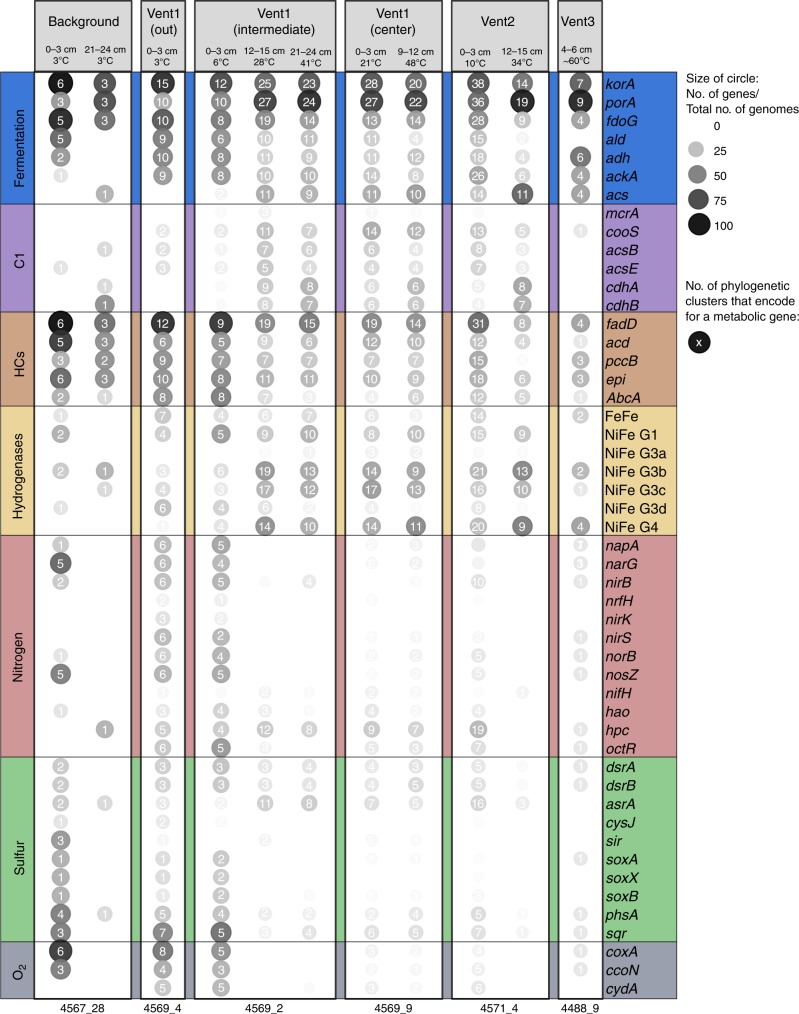
Metabolic profile across different GB sediment sites, depth profiles and temperature regimes. Shown is the number of core metabolic genes relative to the total number of genomes (in %) per site, depth and temperature regime. Temperatures are averages for the 2 or 3 cm thick sediment layers from which DNA was isolated. Background samples: Cold GB samples without hydrothermal activity. Vent1–3: Hydrothermal sediment sampling locations, see also Fig. 1. ID at the bottom: number codes designating every Alvin dive and sediment core (see also Supplementary Data 1 for further explanation). A complete list of metabolic genes can be found in Supplementary Data 10. Number in circles: Number of phylogenetic clusters that encode for individual core metabolic genes at each site

To assess whether hydrothermal sediments not only host a greater phylogenetic but also metabolic diversity than background samples (Fig. 2), we next investigated the spatial distribution of core metabolic genes across all sites and taxa. Regardless of their origin, most genomes encoded genes for general carbon cycling (CAZymes, peptidases, gluconeogenesis, glycolysis), fermentation and lipid oxidation (Fig. 6 and Supplementary Data 10). Respiratory genes were restricted to cooler, shallower samples but present in both background and hydrothermal sediment cores. For example, denitrification genes, SOX genes or the cytochrome c oxidase were found only in the shallower, colder sediments (temperature ~5 °C) and were present in ~20-30% of genomes. In contrast, these genes were represented in only ~0-4% of genomes in deeper, hotter samples (temperature range of 10 °C-60 °C). Exceptions were genes for sulfate/sulfite reduction, such as *dsrAB*, that were still found in ~8% of genomes in deeper, hotter sediments. Compared to background samples, genes involved in C1-metabolism and hydrogenases were more frequently found in hydrothermal sediments. In background sediments only one Bathyarchaeotal genome contained carbon fixation-related genes (*cdhAB*), while genes for methane cycling (*mcrA*) were undetectable. Hydrogenases belonging to Group 4 g, which represent membrane-bound hydrogenases that generate a proton-motive force for energy generation, were absent from the background but present in ~25-30% of genomes across all hydrothermal samples (Fig. 6 and Supplementary Data 10). These findings suggest that methane and hydrogen might be important drivers of metabolic processes in GB hydrothermal sediments.

With few exceptions most metabolic genes were encoded in several taxonomically distinct lineages. For example, C1-related genes (with the exception of *mcrA*) and genes related to beta-oxidation, hydrogen, nitrogen, sulfur and oxygen cycling were found in ~10 different phylogenetic lineages; fermentation genes were present in most phylogenetic clusters of both the archaeal and bacterial community. While the studied genomic dataset from the cold and hydrothermal samples were not represented by an equal number of genomes (average of ~9 and ~60 genomes per habitat type, respectively), we still find that those genomes represent the community well in terms of phylogenetic diversity (Supplementary Figure 4). Additionally, when searching for a subset of these core metabolic genes in binned and unbinned contigs from the complete assembly (only considering contigs > 2,000 bp), we observed a similar trend (Supplementary Data 14). For example, fermentation genes were abundant across all sites, denitrification genes were more common in cold and shallow samples and *mcrA* was completely absent from the background samples. Overall, these findings suggest that the GB genomes are representative of the community as a whole, and that they reflect key metabolic differences between the microbial communities present in hydrothermal and background samples.

## Discussion

In this study, we employed the largest genomic sampling of GB sediments to date to investigate the interplay of community composition and functional diversity. Compared to earlier work on Guaymas Basin sediments^20^, the higher sampling number and inclusion of background samples allowed to better describe the enhanced diversity present in these sediments and shed light on the drivers of community assembly. In contrast to previous studies showing that sulfidic- and methane-rich seep sediments host a lower microbial diversity compared to non-seep marine sediments^39,40^, we demonstrate that GB hydrothermal sediments contain a diverse community that is enriched in archaea compared to a less diverse, bacterial-dominated community found in nearby cold sediments. Therefore, the more extreme conditions in hydrothermal sediments, which include steep thermal and geochemical gradients^17,27^, appear not to inhibit microbial diversity. Due to difficulties in isolating sufficient amounts of DNA from deeper, hotter samples, we cannot exclude that diversity may decline in those sediments. Earlier work reported a decrease in cell numbers with increasing depth that did not necessarily correlate with a decrease in OTU numbers^25^, potentially explaining our difficulties in isolating sufficient amounts of DNA but supporting our assumption that steep temperature gradients do not necessarily inhibit microbial diversity. Especially samples from core 4569_9 experience a highly variable, fluctuating thermal regime over time, where even surficial layers can vary from 20 °C to 70 °C, as determined by multi-day continuous thermal logging (Supplementary Figure 1)^17^. In response to such conditions, microbes must either adapt, have a wide thermal optimum, as shown for some ANME-1 archaea^23^, or be able to recolonize the sediment after a temperature sweep from a surficial reservoir^41^. Here, we propose that the diverse communities inhabiting hydrothermal sediments could serve as a flexible seed bank for the deeper, hotter sediments as well as for highly fluctuating environmental gradients in shallow sediments^5,25,42^.

The differences we observed in community composition across sites were not always translated into obvious changes in functional capacities of those communities. For example, we detected abundant genes for carbon cycling and fermentation across all sites, while other metabolic processes such as respiration, were limited to shallow sediments but present in both background and hydrothermal sediments. Respiratory processes were often partitioned among the community and only few genomes were encoding for full pathways. Metabolic handoffs have been observed in other microbial communities and could allow a flexible interchange of metabolites between changing populations^43,44^. Another metabolic feature that could allow for greater ecosystem stability could be metabolic plasticity, i.e. switching metabolic processes in response to changes in environmental conditions. We found indications for such plasticity in several bacterial genomes, especially within the Delta- and Gammaproteobacteria that might couple the reduction of sulfur with the oxidation of carbon, lipids or hydrocarbons. While we cannot determine which processes are active, enhanced genotypic diversity might provide an additional adaptation strategy to variable environmental conditions.

The only functional categories that were consistently enriched across all hydrothermal sites and almost absent in background sediments were group 4g hydrogenases and pathways for methanogenesis and methane oxidation. Group 4g hydrogenases are not well characterized but are generally described to be membrane-bound hydrogenases that allow for energy-generation by establishing ion gradients over the membrane^45^. These complexes are often found in thermophiles, such as *Pyrococcus furiosus*^45^, and could potentially provide a selective advantage in hydrothermal sediments over other energy-generating systems. While trace concentrations of biogenic methane are present in background sediments (Supplementary Data 1, Supplementary Methods), the inability to detect *mcrA* in these samples could be because of sequencing depth; in contrast detecting *mcrA* in hydrothermal sediments appears to be linked to microbial methane oxidation produced by pyrolysis of organic matter^17^.

Within the phylogenetically and functionally diverse community inhabiting GB, the metabolic repertoire shows a high degree of functional redundancy across different phyla, i.e. different taxa encode the same metabolic function and thus might substitute for one another. Therefore, even if community composition varies, metabolic function is predicted to be relatively stable. Like phylogenetic diversity, functional redundancy could benefit the community when dealing with perturbations in environmental conditions and has been observed in other environments including the global marine or humane microbiome^46,47^. While any stressor, such as temperature, might result in the removal of a given taxon, functional redundancy across different lineages that are each tolerant to some degree of environmental fluctuations, and together cover a wide window of environmental conditions, ensures the stability of community function. This is consistent with the ‘it’s the song not the singer’ (ITSNTS) theory, which assumes that surviving taxa replace perturbed taxa (‘the singers’) and thereby allow nutrient cycles (‘the song’) to persist in the environment^48^. This theory is consistent with our findings, in which we not only observe phylogenetically diverse but also functionally redundant communities. Altogether, the phylogenetic diversity, metabolic partitioning as well as functional redundancy that we observe appear to be characteristics of microbial communities in these dynamic hydrothermal vent sediments.

One question that arises when observing functional redundancy within a microbial community is whether this redundancy enhances species competition and de-stabilizes the community^49,50^. While it is not in the scope of this study to discern niche patterns, we would assume that the high redundancy in our dataset might still allow microbes to inhabit different niches. Two mechanisms that could allow co-existence of supposedly redundant microbes could be metabolic auxotrophies or heterogeneity in limiting resources and/or environmental conditions^50–52^. Amino acid auxotrophies can create community interdependencies, which could balance competition and thereby stabilize microbial communities^53^. We do see indications for such interdependencies in our dataset, where auxotrophies are common in small genomes belonging to CPR bacteria (Supplementary Data 10). Additionally, we assume that the diverse GB-inhabiting communities are stabilized by the high abundance of substrates present in hydrothermal sediments, which might reduce competition and allow taxa to coexist. Finally, while genes for core metabolic processes showed a high redundancy across our dataset, we hypothesize that enzymes involved in substrate degradation are undergoing substantial diversification with respect to their substrate spectra. The diversity of genes involved in carbohydrate (*mcrA*, CAZYmes), lipid (acyl-CoA dehydrogenase) and peptide degradation and the expanding substrate range and diversity of hydrocarbon-degrading genes, such as *mcrA*, supports this notion^16,20,54^. A limitation of the current study that complicates a definite description of the diversity patterns and functional redundancy present in Guaymas sediments is the low sample number and limited number of bins recovered from a subset of samples (i.e. 4567_28 and 4488_9); given the limitations of deep-sea sampling, different habitat and sediment types are represented unevenly. Activity-based analyses of large sample numbers, i.e., metatranscriptomics, would more rigorously link genetic patterns to their environmental determinants.

Guaymas Basin is a hotspot for microbial biodiversity and an ideal study site to investigate the functional diversity of hydrothermally influenced seafloor sediments. Here we establish that these hydrothermal sediments contain a large number of archaeal and bacterial lineages, including several uncultivated phylum-level lineages that have not been described from other habitats. Intriguingly, hydrothermal GB sediments hosted a greater diversity compared to surrounding non-hydrothermal sediments contrasting previous work on methane seep communities^39,40^. These differences are likely linked to the unique environment in GB sediments characterized by by convective mixing of fluids resulting in variable thermal regimes, and admixture of hydrothermal carbon and energy sources. Most functional properties were shared widely among different phylogenetic lineages across different sampling sites with a greater functional redundancy of metabolic processes found in hydrothermal sediments. One unique functional trait of hydrothermal compared to background sediments was the presence of methane cycling genes among novel lineages, including a new deep-branching Crenarchaeota group. We propose that the combination of dynamic seep and hydrothermal conditions in Guaymas Basin enhances microbial diversity, and sustains a distinctive microbial community, whose functional complexity and redundancy reflects the intricate and dynamic geochemical and thermal landscape of this habitat.

## Methods

### Sampling

Guaymas Basin sediment samples were collected from the Gulf of California (27°N0.388, 111°W24.560) at a depth of approximately 2000 m below the water surface. Sediment cores were collected during four Alvin dives (4488, 4569, 4567, and 4571) in 2008 and 2009 (Supplementary Data 1). Sample site photos were compiled from the *Alvin* frame grabber site **(**http://4dgeo.whoi.edu/alvin). Intact sediments were collected during *Alvin* dives using polycarbonate cores (45-60 cm in length, 6.25 cm interior diameter), subsampled into cm layers under N_2_ gas in the ship’s laboratory and immediately frozen at −80 °C. Eleven sediment subsamples for DNA isolation from different depth profiles yielded sufficient genomic DNA for metagenomic sequencing (Supplementary Data 1). Higher temperature samples were tested as well but did not yield sufficient DNA for metagenomic sequencing. Metadata for all dives, including details on the geochemistry (i.e. methane concentrations and dissolved organic carbon concentrations and δ^13^C values, sulfate and sulfide concentrations) and thermal profiles of the sampling sites, are available to compare microbial community composition across sediment cores (Supplementary Data 1, Supplementary Methods)^17^. Additional images and descriptions of the sampling locations are published in a survey of different Guaymas Basin habitats^18^.

### Metagenomic sequencing and assembly

Total DNA from ≥ 10 g of sediment from each of the eleven samples (see above) was extracted using the MoBio PowerMax soil kit using the manufacturer’s instructions. DNA concentrations were measured using a Qubit™ 3.0 Fluorometer and a final concentration of 10 ng/µl of each sample (using a total amount of 100 ng) was used to prepare libraries for paired-end Illumina (HiSeq–2500 1TB) sequencing. Illumina library preparation and sequencing was performed at the Joint Genome Institute (JGI). Sequencing was performed on an Illumina HiSeq 2500 machine using the paired end 2 × 125 bp run-type mode. All runs combined provided a total of ~280 gigabases of sequencing data (Supplementary Data 2) Quality control and sequence assembly was performed by JGI. Briefly, sequences were trimmed and screened for low quality sequences using bbtools (https://jgi.doe.gov/data-and-tools/bbtools/) and assembled using megahit v1.0.6 using the following options: --k-list 23,43,63,83,103,123^55^. Summary statistics for the number of generated reads and the quality of the metagenomic assembly is provided in Supplementary Data 2. For further binning, only scaffolds ≥ 2000 bp were included.

### Metagenomic binning

Metagenomic binning was performed on individual assemblies using the binning tools ESOM, Anvi’o and Metabat. ESOM binning was performed by calculating tetranucleotide frequencies of scaffolds with a minimum length of 2000 bp using the K-batch algorithm for training after running the perl script esomWrapper.pl^56^. The resulting Emerging Self-Organizing Maps (ESOM) were manually sorted and curated. Bins were extracted using getClassFasta.pl (using -loyal 51). The binning process was enhanced by incorporating reference genomes as genetic signatures for the assembled contigs into ESOM. For Anvi’o (v2.2.2) the metagenomic workflow pipeline that incorporates CONCOCT was used for binning^57^. Briefly, coverage information was obtained by generating eleven mapping files for each assembly file by mapping all high-quality reads of each of the eleven samples against the assembly of one sample using the BWA-MEM algorithm in paired-end mode (bwa-0.7.12-r1034; using default settings)^58^. The resulting sam file was sorted and converted to bam using samtools (version 0.1.19)^59^. The bam file was prepared for Anvi’o using the script anvi-init-bam and a contigs database generated using anvi-gen-contigs-database. These two files were further used as input for anvi-profile. Generated profiles for the eleven different assemblies were combined using anvi-merge and the resulting bins summarized using anvi-summarize (-C CONCOT). If not mentioned otherwise, the scripts were used with default settings. Finally, binning was performed using metabat (v1)^60^. As described for Anvi’o the used input files consisted of the scaffold files (≥2000 bp) and the mapping files to recover bins both by sequence composition and abundance across samples. First, each of the mapping files were summarized using jgi_summarize_bam_contig_depths and then metabat was run using the following settings: --minProb 75 --minContig 2000 --minContigByCorr 2000. Results from the three different binning tools were combined using DAS Tool (version 1.0)^61^. Therefore, for each of the binning tools a scaffold-to-bin list was prepared and DAS Tool run on each of the eleven scaffold files as follows: DAS_Tool.sh -i Anvio_contig_list.tsv,Metabat_contig_list.tsv,ESOM_contig_list.tsv -l Anvio,Metabat,ESOM -c scaffolds.fasta --write_bins 1.

The accuracy of the binning approach was evaluated by calculating the percentage of completeness and contamination using CheckM lineage_wf (v1.0.5; Supplementary Data 3)^62^. Genomes were only analyzed further if they were more than 50% complete and showed a contamination below 10%. Contaminants that were identified based on their phylogenetic placement (wrong taxonomic assignment compared to the average taxonomic assignment of the genes assigned to each bin), GC content (>25% difference compared to the mean of all scaffolds assigned to each bin) or abundance (>25% differences compared to the mean abundance of all scaffolds assigned to each bin) were manually removed from individual genomes. This yielded a total of 247 archaea and 304 bacterial genomes.

### Relative abundance

To determine the relative abundance of each genome across the elven sequenced sediment samples, we mapped the contigs from all binned genomes (i.e., using the “whole MAG”) against the high-quality reads of each individual metagenome (generating eleven sam files). The sam output was sorted and converted to bam as described above and we then used the metabat output, which describes the read counts recruited by each contig, for further analyses. All analyses were performed in R (version 3.3.3). On average, ~47% of the high-quality metagenomic sequences could be binned, with the notable exception of the sample from 4567_28, from which the recovered MAGs only recruited ~18% of reads for an undetermined reason.

To determine the average abundance of major taxonomic groups (referred to as cluster, which were determined by the phylogenetic analysis described below), contigs were first assigned to their phylogenetic cluster (see description for the phylogenetic analysis below) and were then summarized using the ddply function from the plyr package^63^. These clusters do not represent a specific taxonomic rank but were chosen to account for both phylogenetic diversity (i.e., Crenarchaeota are usually represented at order rank or lower if possible) as well as available genomes (the different phyla of the CPR superphylum were ranked together because they were represented by only few genomes). The counts recruited by each taxonomic group were normalized by the total length of contigs belonging to each cluster, the library size of the individual metagenomes and multiplied by 1000 for better readability. The normalized relative abundance was plotted using the heatmap.2 function in the gplots package. The summary statistics are provided in Supplementary Data 7 (only includes clusters with ≥3 lineages).

To determine the relative abundance of the ribosomal protein S3 (RPS3) across samples, RPS3 was extracted from all eleven assemblies (only considering contigs >2000 bp) using phylosift (v1.0.1 using options: phylosift all --keep_search --custom marker_list.txt). In total, we identified 1227 RPS3 sequences in the dataset, 486 of which belonged to binned contigs (~40%) and, therefore, RPS3 could be successfully recovered from ~82% of bins; (Supplementary Table S4). The unaligned nucleotide sequences were concatenated and used as an input to run bwa against all eleven metagenomes to determine their relative abundance across samples. Read counts were extracted using samtools, normalized by gene length and library size and plotted using the *ggplot2* package in R.

### Phylogenetic analyses

Phylosift was used to extract marker genes for the phylogenetic placement of the assembled metagenomic bins^64^. A set of 37 single-copy, protein-coding housekeeping genes was chosen for a further phylogenetic analysis (Supplementary Data 4). To generate a reference dataset, archaeal (all available genomes) and bacterial genomes (selected genomes that include at least three members from each genus and a preference for type strains whenever possible) were downloaded from NCBI on March 2017. Next, all reference genomes and GB genomes (fasta files) were used as an input for phylosift (v1.0.1) using the ‘phylosift search’ followed by the ‘phylosift align’ mode. The concatenated protein alignments of 37 elite marker genes (concat.updated.1.fasta) were combined for all genomes of interest and trimmed using TrimAL (version 1.2) using the automated1 setting^65^. A phylogenetic tree was generated using a maximum likelihood-based approach using RAxML (version 8.2.10, called as: raxmlHPC-PTHREADS-AVX -f a -m PROTGAMMAAUTO -N autoMRE)^66^. The tree was visualized using the Interactive Tree Of Life (iTOL) webtool^67^. For better visualization, the initial tree was reduced to only include references that were branching close to GB genomes and included a 224 genomes from cultured representatives and 330 genomes from uncultured genomes (including metagenome-assembled genomes, enrichment cultures, co-cultures and single-cell assembled genomes). All of these genomes were used to calculate an average amino acid identity across all genomes using comparem (v0.0.23, function aai_wf; https://github.com/dparks1134/CompareM). The AAI was used as a main measure to distinguish the new phylum-level genomes that were discovered with the phylogenetic approach. Therefore, the average AAI of each phylum was calculated and compared to all remaining phyla, especially those branching close to the phyla of interest (see also Supplementary Data 6).

The 16S rRNA gene sequences were extracted using phylosift (settings are described above) and barrnap (https://github.com/tseemann/barrnap, v0.7, settings: --kingdom arc/bac --lencutoff 0.2 --reject 0.3 --evalue 1e-05) and aligned to the SILVA SSURef_NR99 database (release 13.12.2017) using the SILVA webaligner^68^. The alignment was manually curated in ARB. A phylogenetic tree was generated using a maximum likelihood-based approach using RAxML (settings: raxmlHPC-PTHREADS-AVX -T 10 -f a -m GTRGAMMA -N autoMRE -p 12345 -x 12345). 16S rRNA gene sequences were manually checked for contamination in cases with an inconsistent phylogenetic assignment between 16S rRNA gene sequences or the 37 protein-coding marker genes. The whole contig was discarded, when all assigned proteins on the contig with the 16S rRNA gene showed a different taxonomic assignment (using blastp) compared to the remaining scaffolds of the respective genome.

A similar phylogenetic approach was taken to phylogenetically characterize other key genes of interest (i.e. hydrogenases, *mcrA*, glycyl radical enzymes, acyl-CoA dehydrogenases (*acd*)). Genes of interest were identified in GB genomes using KAAS, HMMER, blastp or the HydDB webserver (for details see below). Published reference genes were extracted using the NCBI and Uniprot webservers (McrA, glycyl radical enzymes, ACD) as well as the HydDB webserver (hydrogenases). For the glycyl radical enzymes, proteins identified as PflA, AssA, BssA, HbsA, MasD, NmsA in KEGG or a custom blast search were combined in a single analysis. For the ACD phylogeny, the KAAS IDs K00248, K00249, K06445, K00255, K06446 and K09479 were included to build a phylogenetic tree. Protein sequences from GB and reference genomes were combined and aligned using muscle (v3.8.31, default settings), trimmed using TrimAL and a phylogenetic tree generated using RAxML as described above. Protein-coding genes falling on long branches were manually checked using blastp on the NCBI webserver and discarded if the annotation was not hydrogenase, acyl-CoA dehydrogenase or glycyl radical enzyme.

### Annotations and metabolic analyses

Gene prediction for individual genomes was performed using prodigal (V2.6.2, default settings)^69^. The genomes contained on average 1,665 predicted proteins for archaea (min = 500 and max = 4,685) and 2,491 for bacteria (min = 636 and max = 6,964) (Supplementary Data 3). Metabolic reconstructions were done for each individual genome, but in several cases the results were summarized for major taxonomic lineages, or clusters. These clusters do not represent a specific taxonomic rank but were chosen to account for both phylogenetic diversity (Crenarchaeota are usually represented at order rank) as well as available genomes (the different phyla of the CPR superphylum were ranked together because they were represented by only few genomes).

Predicted genes of individual genomes were further characterized using KAAS (KEGG Automatic Annotation Server; Supplementary Data 10)^70^. Therefore, protein sequences of each of the individual genomes were uploaded to the KAAS webserver using the ‘Complete or Draft Genome’ setting (used parameters: GHOSTX, custom genome dataset, BBH assignment method). For a detailed pathway analysis the KO numbers were downloaded, concatenated and merged with a KO-to-pathway metadata file in R (Supplementary Data 10).

Additionally, we searched for key metabolic genes using custom blastp and hmmer databases^43^. A Curated Database of Anaerobic Hydrocarbon Degradation Genes (AnHyDeg) and the MEROPS database were used to identify hydrocarbon degradation genes as well as peptidases in the concatenated proteins sequences of all GB genomes using blastp (e-value threshold of 1e-20; Supplementary Data 10)^71–73^. Hits were discarded if they were related to core metabolic processes (i.e., pyrimidine synthesis) or included heat-shock resistance proteins, precursor proteins and signal peptides. Additionally, we utilized a custom hmmer as well as the Pfam and TIGRFAM databases to search for key metabolic marker genes using hmmsearch and custom bit-score cutoffs^43,74,75^. Hydrogenases were extracted from the genomes using hmmsearch (e-value cut-off of 1e-20) and confirmed using a web-based search using the hydrogenase classifier HydDB^76^. Finally, genes encoding for carbohydrate degradation enzymes described in the Carbohydrate-Active enZYmes (CAZYmes) database were identified using the dbcan webtool and applying an e-value threshold of 1e-5^77^. Protein localization was determined for CAZYmes and peptidases using the command-line version of Psort (V3.0) using the option --archaea for archaeal genomes. The results for the MEROPS and CAZymes database searches are summarized in Supplementary Data 8 and 9. In the case of protein-coding genes hitting to multiple genes in the before-mentioned databases, the best hit was chosen based on their e-value and bit-score using blast_best.pl (http://alrlab.research.pdx.edu/aquificales/scripts/).

Genes assigned to core metabolic pathways are summarized in Supplementary Data 10. Hits for key metabolic marker genes found in major taxonomic clusters (Fig. 3) were verified across different databases (KAAS, PFAM and TIGRPFAMs) and cross-checked with results from close reference genomes that fell within the same phylogenetic group as the genome of interest to reduce the chance of contamination. Genes not found in close reference genomes were further validated with blastp using the NCBI webserver tool. If a hit could not be confirmed or if the top phylogenetic hit for whole contig was not consistent with the phylogenetic assignment of the genome, it was removed from the genome.

## Electronic supplementary material

Description of Supplementary Files

Supplementary Information

Supplementary Data 1

Supplementary Data 2

Supplementary Data 3

Supplementary Data 4

Supplementary Data 5

Supplementary Data 6

Supplementary Data 7

Supplementary Data 8

Supplementary Data 9

Supplementary Data 10

Supplementary Data 11

Supplementary Data 12

Supplementary Data 13

Supplementary Data 14

Peer Review File

## Data Availability

All sequence data and sample information are available at NCBI under BioProject ID PRJNA362212. Accession numbers for individual genomes can be found in Supplementary Data 3. Additionally, the raw data is provided in IMG/MER and the IMG Genome IDs for the individual metagenomes are provided in Supplementary Data 1.
