# Supplementary material for: Expansive microbial metabolic versatility and biodiversity in dynamic Guaymas Basin hydrothermal sediments
Source: Nat Commun. 2018 Nov 27;9:4999. doi: 10.1038/s41467-018-07418-0 (PMC6258724; doi:10.1038/s41467-018-07418-0)
Supplement: Supplementary file 1 — Description of Supplementary Files [file 41467_2018_7418_MOESM1_ESM.docx]

**Description of Supplementary Files**

**Supplementary Information:** Supplementary information including extended methods and Supplementary Figures.

**Supplementary Data 1:** Sample metadata. Metadata for Guaymas Basin hydrothermal sediment samples collected from dives 4569 (Vent1), 4567 (Vent2) and 4488 (Vent3). Samples from nearby cold, background sediments were collected from dive 4567. For Vent1, three sediment cores were taken inside the yellow mat (4569_9), further outside in a white mat area (4569_2) and outside of the mat area (4569_4); next to each core a thermal logging probe was inserted into the sediment. At Vent2 and Vent3, one core each (4571_4 and 4488_9) was sampled. Beggiatoa refers to the presence or absence of a mat of Gammaproteobacteria and the respective color of the mat. Asterisk: Due to low amounts of DNA these samples were processed with the ultra-low Nextera protocol.

**Supplementary Data 2:** Assembly statistics. Statistics for the sequence assembly generated from Guaymas Basins sediment samples. High-quality reads were obtained after filtering for potential contamination (i.e. eukaryotic sequences, adaptor sequences), removing low quality reads (phred score < 10) and reads that were too short (reads < 100 bp). Binned metagenomes (%) refers to the percentage of high-quality bins mapped against the binned genomes (for total numbers please refer to Supplementary Data 7).

**Supplementary Data 3:** Genome statistics. Summary information for all genomes binned from Guaymas Basin sediment samples. Includes information on the taxonomy (determined by a phylogenetic analysis of 37 marker genes), genome statistics and completeness estimates (determined by CheckM). Number of single-copy marker genes with no hit (0) or up to 5 hits (5). If a 16S rRNA gene sequence was present in a genome this is highlighted with a y. The relative abundance was determined by mapping each genome against each of the eleven metagenomes using BWA with default settings.

**Supplementary Data 4:** Number of phylogenetic marker genes found in individual GB genomes. Number of identified marker genes that were used to phylogenetically assign each genome (determined using phylosift). The DNGNGWU marker gene in phylosift refers to a suite of single-copy, protein-coding marker genes. Count Markers: Total number of hits for each genome (not accounting for doubletons).

**Supplementary Data 5:** Tree file for the maximum likelihood phylogenetic tree of GB genomes based on 37 concatenated protein-coding genes shown in Figure 2 and Supplementary Figure 2.

**Supplementary Data 6:** Average amino acid identity of GB and reference genomes. Average amino acid identity (AAI) in percent of all GB genomes compared to the reference genomes depicted in Supplementary Figure 2. Highlighted are values >= 50%. Source: Source of genomic sequence, which allows to discriminate between genomes with cultured and uncultured origin. The order in the table reflects the order of the tree provided in Supplementary Figure 1, therefore, genomes with a close phylogenetic distance are next to their closest neighbor in the table.

**Supplementary Data 7:** Read counts recruited by major taxonomic lineages across Guaymas Basin sediment samples. Summarizes the results of read mapping of all 551 GB genomes against the raw sequence data using BWA. The total number of reads recruited by genomes belonging to the same taxonomic cluster were summed to calculate the relative abundance of each lineage across each of the sampled sediments. This analysis does not include clusters with less than three genomes.

**Supplementary Data 8:** Total number of carbohydrate-active enzymes (CAZymes) detected in each GB genome. Total number of identified auxiliary activities (AAs), carbohydrate-binding modules (CBMs), carbohydrate esterases (CE), glycoside hydrolases GH) and polysaccharide lyases (PL). Subcellular localization for CEs, GHs and PLs was determined using PSORT. Also provides the raw output from the dbCAN webserver and PSORT results (sheet ‘raw’).

**Supplementary Data 9:** Total number of peptidases detected in each GB genome. Total number of peptidases identified using the MEROPS peptidase database. Subcellular localization for individual peptidases was determined using PSORT. Also provides the raw output from the blastp search against the MEROPS peptidase database and PSORT results (sheet ‘raw’).

**Supplementary Data 10:** Summary of metabolic genes encoded within each GB genome. Total number of metabolic genes identified in individual GB genome using the KAAS webserver, custom HMMER searches and blastp and the HydDB webserver (used database is indicated in the column DB). Count: Summarizes the total hits for each metabolic genes (not accounting for doubletons). Genes were ordered based on their occurrence in metabolic processes. Core genes: Core metabolic genes used to generate Figures 4 and 6. AssAD: Notice, here several genes belonging to the glycyl-radical enzyme family were combined as they do share a high sequence similarity that is difficult to differentiate based on a blastp search alone; therefore a detailed phylogeny is provided in Supplementary Figure 7.

**Supplementary Data 11:** Tree file for the maximum likelihood phylogenetic tree of the methyl-Coenzyme M reductase protein detected in GB genomes shown in Figure 5.

**Supplementary Data 12:** Tree file for the maximum likelihood phylogenetic tree of acyl-CoA dehydrogenases detected in GB genomes shown in Supplementary Figure 7.

**Supplementary Data 13:** Tree file for the maximum likelihood phylogenetic tree of glycyl radical enzymes detected in GB genomes shown in Supplementary Figure 8.

**Supplementary Data 14:** Summary of core metabolic genes found across GB assemblies. Total number of core metabolic genes identified in assemblies from different GB sediments (considering only scaffolds > 2,000 bp). Core metabolic genes were searched using their KO term or EC number in JGI annotation data. Genes were ordered based on core metabolic processes that reflect processes shown in Figure 6. Number below the sample ID reflects the total number of proteins detected in that metagenome (excluding any annotations with the term ‘hypothetical protein’).
