# Supplementary material for: Expansive microbial metabolic versatility and biodiversity in dynamic Guaymas Basin hydrothermal sediments
Source: Nat Commun. 2018 Nov 27;9:4999. doi: 10.1038/s41467-018-07418-0 (PMC6258724; doi:10.1038/s41467-018-07418-0)
Supplement: Supplementary file 2 — Supplementary Information [file 41467_2018_7418_MOESM2_ESM.pdf]

Supplementary Information

Expansive microbial metabolic versatility and biodiversity in dynamic Guaymas Basin hydrothermal sediments

Dombrowski et al.

Supplementary Methods

Geochemical and thermal profiles were determined as described previously[1].Briefly, thermal gradients were measured by a heatflow probe before pushcore sampling; geochemical gradients were determined after sediment subsampling and porewater extraction from freshly recovered cores on the ship, typically within 4-6 hours after recovery. For sampling at "vent1" and "vent2" (termed "marker 14 mat" and "T-logger site 3" in ref.[1]), the heatflow probe measurements were supplemented by week-long thermal logger deployments (Supplementary Figure 1) after recording the initial temperature profiles given here. The sediment cores were collected together with the thermal logging probes 7 to 8 days later[1]. Most geochemical data are included in the Supplementary Material of ref. [1], and they are included here for easy reference; the data for core 4488_10 and the sulfide concentration data are compiled here.

Temperature profiles in °C

	Vent 1<br>4569_9	Vent 1<br>4569_2	Vent 1<br>4569_4	Background<br>4567_28
0 cm	13.3	3.6	3.3	3.1
10 cm	48.0	20.8	7.0	3.2
20 cm	65.3	38.7	13.2	3.4
30 cm	83.4	52.1	18.9	3.6
40 cm	93.7	62.3	23.1	3.8
50 cm	no data	no data	no data	4.1

	Vent 2<br>4571_4	Vent 3<br>4488_9
0 cm	6.9	43
10 cm	27.7	no data
20 cm	43.7	no data
30 cm	55.0	150
40 cm	63.2	no data

Dissolved CH_4 in mM

	4569_4	4569_2	4569_9	4571_4	4567_28
supernat.	0.01	0.09	no data	0.12	>0.01
0-3 cm	0.01	2.86	1.08	2.98	>0.01
3-6 cm	0.03	2.99	2.42	3.16	>0.01
6-9 cm	0.06	2.77	2.32	3.17	>0.01
9-12 cm	0.11	2.34	1.93	3.13	>0.01
12-15 cm	0.20	2.33	1.67	3.59	>0.01
15-18 cm	0.28	2.44	1.61	2.99	>0.01
18-21 cm	0.55	2.30	1.96	2.74	>0.01
21-24 cm	0.78	2.32	2.33	2.39	>0.01
24-27 cm	0.80	2.38	2.05	1.71	no data
27-30 cm	0.84	2.65	2.39	1.65	no data
30-33 cm	0.81	2.64	2.44	1.76	no data
33-36 cm	0.79	2.58	no data	1.51	no data
36-39 cm	0.71	2.61	no data	1.62	no data
39-42 cm	0.71	2,62	no data	1.71	no data

4488_10 [next to 4488_9]	
0-2 cm	2.60
2-4 cm	2.19
4-6 cm	3.92
6-8 cm	2.78
8-10 cm	no data
10-12 cm	2.60
12-14 cm	1.77
14-16 cm	1.48
16-18 cm	no data

$\delta^{13}C\text{-}CH_4$ in ‰

	4569_4	4569_2	4569_9	4571_4	4567_28
supernat.	no data	no data	no data	no data	no data
0-3 cm	-47.6	-38.61	-27.41	-34.32	-68.7
3-6 cm	-42.6	-37.33	-31.02	-35.62	-67.5
6-9 cm	-40.2	-32.12	-29.00	-37.33	-70.2
9-12 cm	-38.5	-28.21	-27.35	-33.60	-69.8
12-15 cm	-35.6	-27.62	-26.95	-34.72	-69.4
15-18 cm	-32.9	-28.00	-34.03	-33.94	-74.2
18-21 cm	-29.1	-26.30	-37.21	-26.96	-69.7
21-24 cm	-28.5	-25.52	-35.72	-16.97	-70.0
24-27 cm	-28.3	-26.32	-36.41	-16.90	no data
27-30 cm	-28.4	-29.63	-37.89	-30.55	no data
30-33 cm	-28.7	-31.9	-37.69	-16.82	no data
33-36 cm	-28.6	-33.00	no data	-15.99	no data
36-39 cm	-28.4	-34.50	no data	-15.80	no data
39-42 cm	-28.1	-35.10	no data	-16.50	no data

4488_10 [next to 4488_9]	
0-2 cm	-39.54
2-4 cm	-39.09
4-6 cm	-39.70
6-8 cm	-39.71
8-10 cm	-39.09
10-12 cm	-39.88
12-14 cm	-40.07
14-16 cm	-39.70
16-18 cm	no data

Porewater Sulfate in mM

	4569_4	4569_2	4569_9	4571_4	4567_28
supernat.	23.66	25.17	23.94	26.57	26.31
0-3 cm	24.25	21.76	22.67	21.85	24.66
3-6 cm	24.22	20.87	22.94	19.94	26.09
6-9 cm	24.94	19.67	22.21	19.36	25.34
9-12 cm	24.31	22.01	23.66	17.24	24.58
12-15 cm	24.20	21.40	22.86	19.17	25.92
15-18 cm	23.48	19.68	23.94	17.62	23.49
18-21 cm	23.25	no data	23.64	14.17	23.53
21-24 cm	24.79	19.00	23.64	11.44	23.61
24-27 cm	24.32	20.94	no data	9.80	no data
27-30 cm	25.75	21.25	22.57	10.28	no data
30-33 cm	26.50	21.95	23.21	10.49	no data
33-36 cm	26.10	no data	no data	10.30	no data
36-39 cm	no data	no data	no data	5.96	no data
39-42 cm	no data	no data	no data	10.93	no data

4488_10 [next to 4488_9]	
0-2 cm	4.28
2-4 cm	10.47
4-6 cm	3.39
6-8 cm	1.22
8-10 cm	0.33
10-12 cm	0.36
12-14 cm	0.78
14-16 cm	1.10
16-18 cm	1.32

Porewater Sulfide in mM

	4569_4	4569_2	4569_9	4571_4	4567_28
supernat.	0.081	0.009	0.131	0.190	0.011
0-3 cm	0.055	0.476	0.881	2.158	0.032
3-6 cm	0.077	1.524	1.396	3.748	0.058
6-9 cm	0.051	1.623	1.569	2.986	0.026
9-12 cm	0.031	2.187	1.907	3.858	0.093
12-15 cm	0.083	2.107	0.991	2.205	0.096
15-18 cm	0.272	2.644	0.947	2.352	0.064
18-21 cm	0.485	2.545	0.706	2.817	0.037
21-24 cm	0.592	2.902	0.776	4.242	0.000
24-27 cm	0.656	2.702	no data	4.295	no data
27-30 cm	0.665	2.384	no data	3.699	no data
30-33 cm	0.606	2.346	no data	3.154	no data
33-36 cm	0.536	no data	no data	3.138	no data
36-39 cm	no data	no data	no data	3.140	no data
39-42 cm	no data	no data	no data	3.192	no data

4488_10 [next to 4488_9]	
0-2 cm	0.27
2-4 cm	3.26
4-6 cm	3.58
6-8 cm	2.70
8-10 cm	2.01
10-12 cm	0.60
12-14 cm	0.96
14-16 cm	0.35
16-18 cm	0.11

Porewater DIC in mM

	4569_4	4569_2	4569_9	4571_4	4567_28
supernat.	3.51	3.32	5.64	4.06	no data
0-3 cm	4.06	10.50	5.06	10.20	3.64
3-6 cm	4.46	16.21	13.64	4.65	5.49
6-9 cm	6.19	18.67	7.32	16.40	3.15
9-12 cm	4.30	19.63	12.01	14.51	4.93
12-15 cm	4.78	16.58	7.17	13.93	4.02
15-18 cm	6.73	17.49	11.72	14.42	4.02
18-21 cm	8.06	20.23	6.15	18.27	6.01
21-24 cm	14.64	17.46	6.99	20.45	6.20
24-27 cm	9.31	17.61	6.54	22.40	no data
27-30 cm	9.05	17.88	24.64	19.76	no data
30-33 cm	6.83	19.06	6.65	20.39	no data
33-36 cm	7.40	16.08	no data	23.18	no data
36-39 cm	no data	no data	no data	19.38	no data
39-42 cm	no data	no data	no data	19.65	no data

4488_10 [next to 4488_9]	
0-2 cm	10.47
2-4 cm	12.57
4-6 cm	13.85
6-8 cm	15.27
8-10 cm	10.10
10-12 cm	9.81
12-14 cm	8.46
14-16 cm	6.95
16-18 cm	5.93

δ^{13}C-DIC in ‰

	4569_4	4569_2	4569_9	4571_4	4567_28
supernat.	-2.29	-4.12	-4.03	-3.11	-3.32
0-3 cm	-4.85	-13.3	-15.37	-19.21	-3.17
3-6 cm	-3.81	-15.79	-17.09	-21.69	-3.56
6-9 cm	-2.4	-18.38	-17.41	-20.37	-4.82
9-12 cm	-4.48	-20.1	-18.28	-22.29	-6.17
12-15 cm	-6.95	-19.72	-16.21	-19.97	-7.98
15-18 cm	-7.95	-21.27	-15.66	-21.21	-8.94
18-21 cm	-9.26	-23.23	-14.68	-23.85	-8.76
21-24 cm	-10.59	-23.48	-13.55	-24.82	-10.25
24-27 cm	-11.06	-22.61	-15.59	-24.97	no data
27-30 cm	-11.8	-21.88	-14.22	-25.07	no data
30-33 cm	-12.13	-20.52	-13.01	-25.08	no data
33-36 cm	-11.15	-20.09	no data	-24.84	no data
36-39 cm	no data	no data	no data	-24.8	no data
39-42 cm	no data	no data	no data	-24.33	no data

4488_10 [next to 4488_9]	
0-2 cm	-2.98
2-4 cm	-6.41
4-6 cm	-5.41
6-8 cm	-4.32
8-10 cm	-4.55
10-12 cm	-3.74
12-14 cm	-5.50
14-16 cm	-3.55
16-18 cm	-3.45

Supplementary Figures

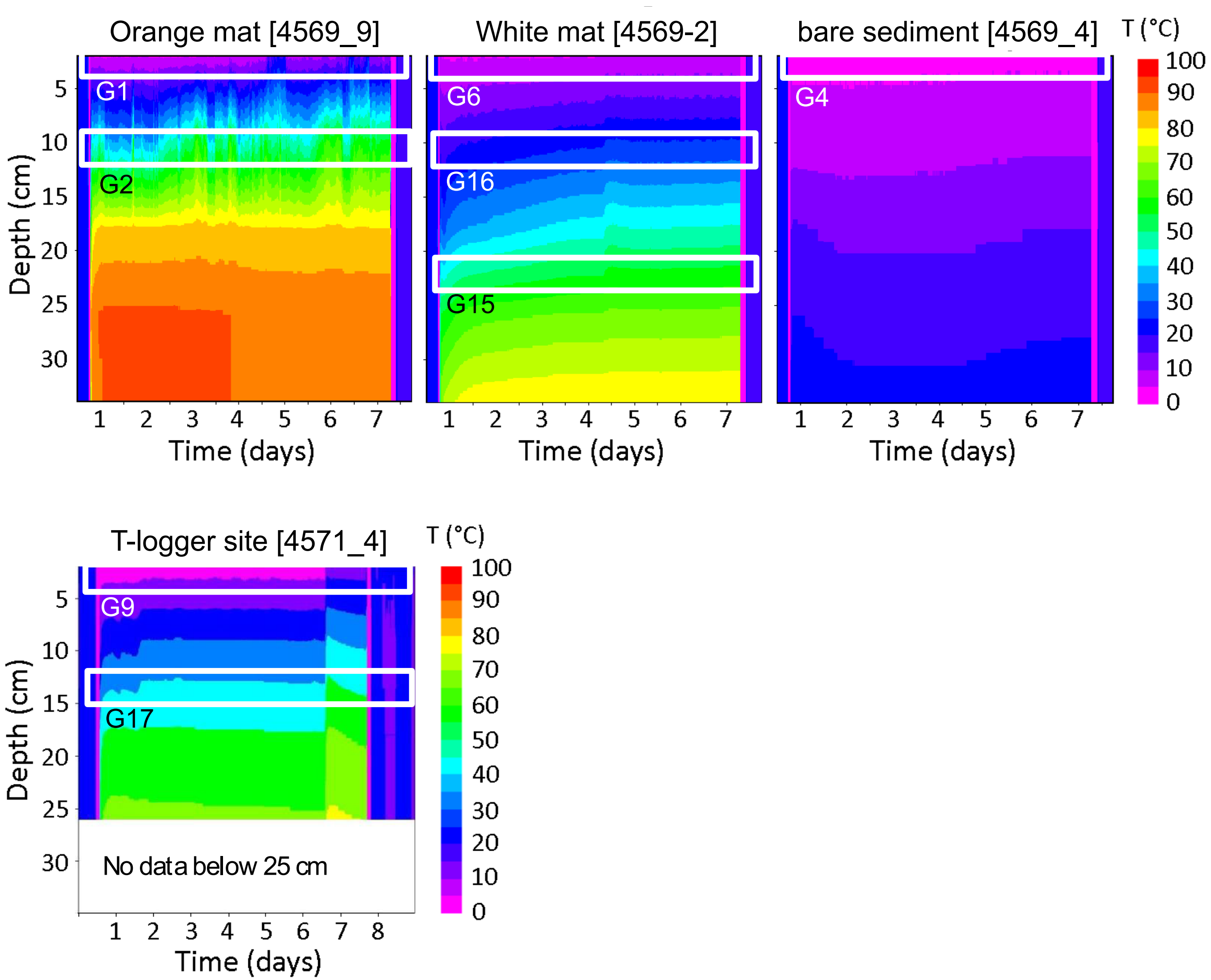

Supplementary Figure 1: Temperature variability in GB sediments. Thermal logging profiles for a core transect through a CH_4/sulfide-rich hydrothermal sediment with orange and white Beggiatoaceae mats, and adjacent bare sediment, all collected during dive 4569 (top), and a nearby hydrothermal sediment with sulfur precipitates collected during dive 4571 (bottom). The shift in temperature after day 6 coincides with the sinking of the temperature logger by ca. 3 cm (bottom). White rectangles: sediment layers selected for DNA extraction and sequencing and identified by sample number as indicated in Supplementary Data 1. Adapted from McKay et al. 2016. Environ. Microbiol.Reports 8:150-161.

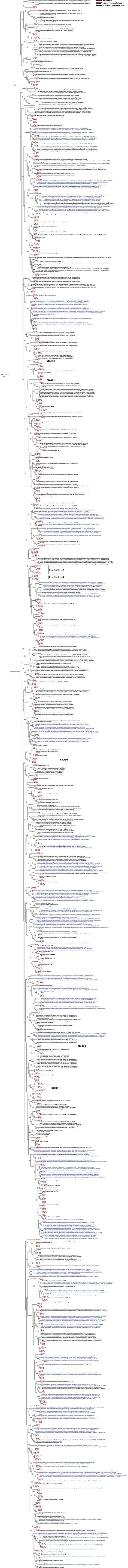
GB Genomes
Cultured representatives
Uncultured representatives
Tree scale: 0.1
GB-AP2
GB-AP1
DeepCrenGroup 1
DeepCrenGroup 2
GB-BP2
GB-BP3
GB-BP1

Supplementary Figure 2: Maximum likelihood phylogenetic tree of GB genomes based on 37 concatenated single-copy, protein-coding genes. Red: Genomes assembled from hot, hydrothermal sediments. Blue: Reference genomes from type material. Black: Reference genomes from uncultured microbes (including metagenomes, enrichment cultures, co-cultures or single-cells). Grey circles: Bootstrap support ≥ 70% (number of bootstraps determined using the extended majority-rule consensus tree criterion). RaxML was run as raxmlHPC-PTHREADS-AVX -f a -m PROTGAMMAAUTO -N autoMRE. The tree file is available in Supplementary Data 5.

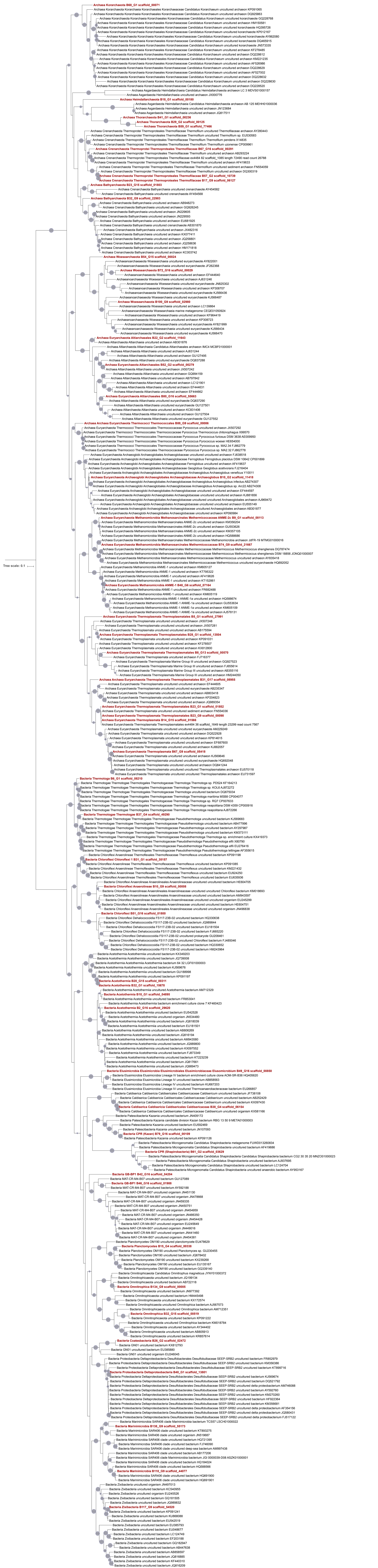
Tree scale: 0.1

Supplementary Figure 3: Maximum likelihood phylogenetic tree of 16S rRNA genes found in GB genomes. Phylogenetic analysis of 16S rRNA gene sequences recovered from GB genomes. Red: GB genomes and the name denotes their phylogenetic assignment as determined using 37 protein-coding marker genes (see also Supplementary Figure 2). Grey circle: Bootstrap support ≥ 70% (number of bootstraps determined using the extended majority-rule consensus tree criterion). RaxML was run as raxmlHPC-PTHREADS-AVX -f a -m GTRGAMMA -N autoMRE.

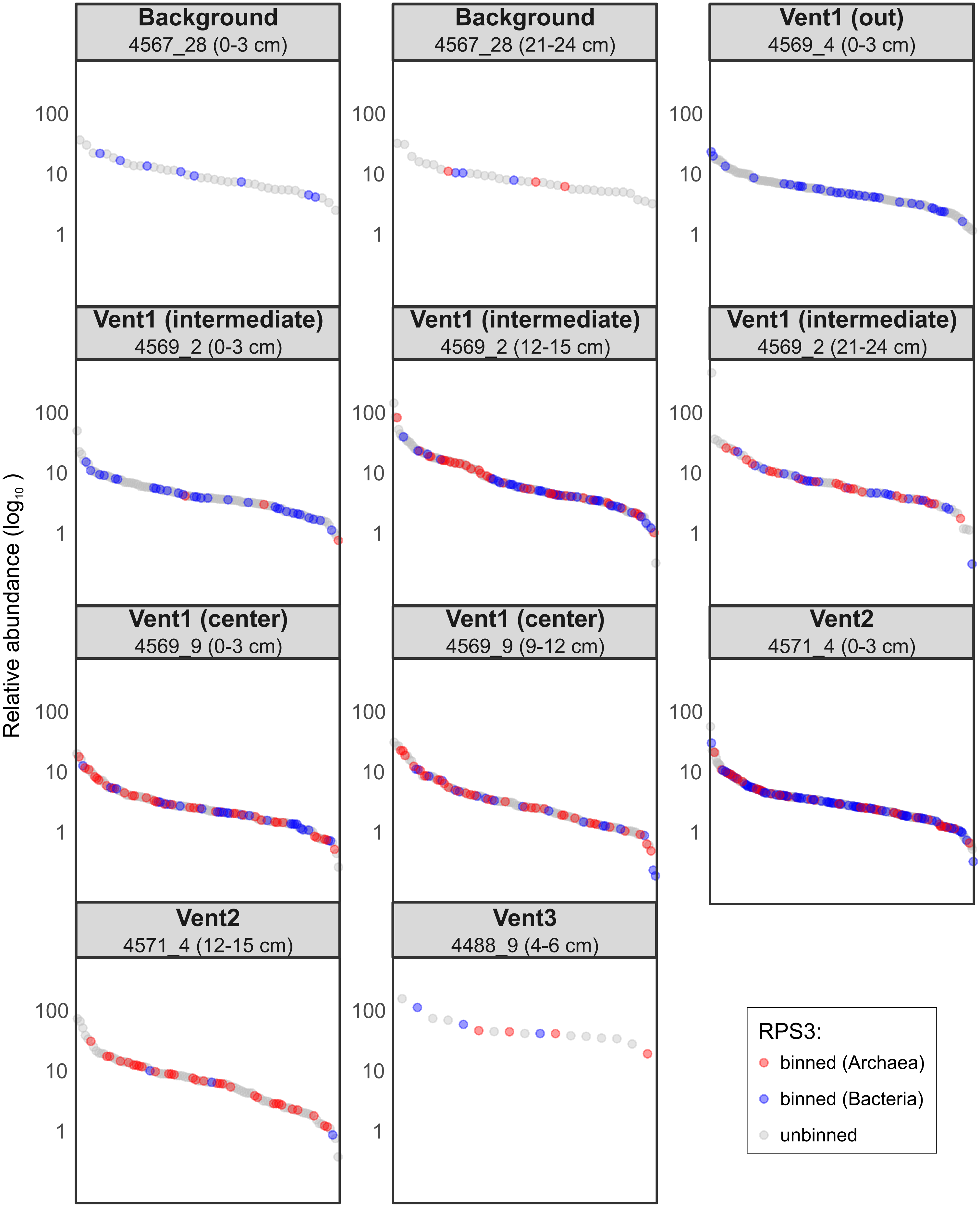

Supplementary Figure 4: Relative abundance of ribosomal protein S3 (RPS3) across GB sediments. Occurrence of RPS3 across assemblies within each GB sediment (normalized by gene length and total library size). x-Axis: Rank order of detected RPS3 proteins ($\log_{10}$-transformed). Grey: Unbinned RPS3 genes. Red, Blue: Binned RPS3 genes from archaeal or bacterial genomes, respectively. Background: Cold GB samples without hydrothermal activity. Vent1-3: Hydrothermal sediment sampling locations, see also Fig. 1. ID below sample name: Dive ID and depth, see also Supplementary Data 1 for further explanation.

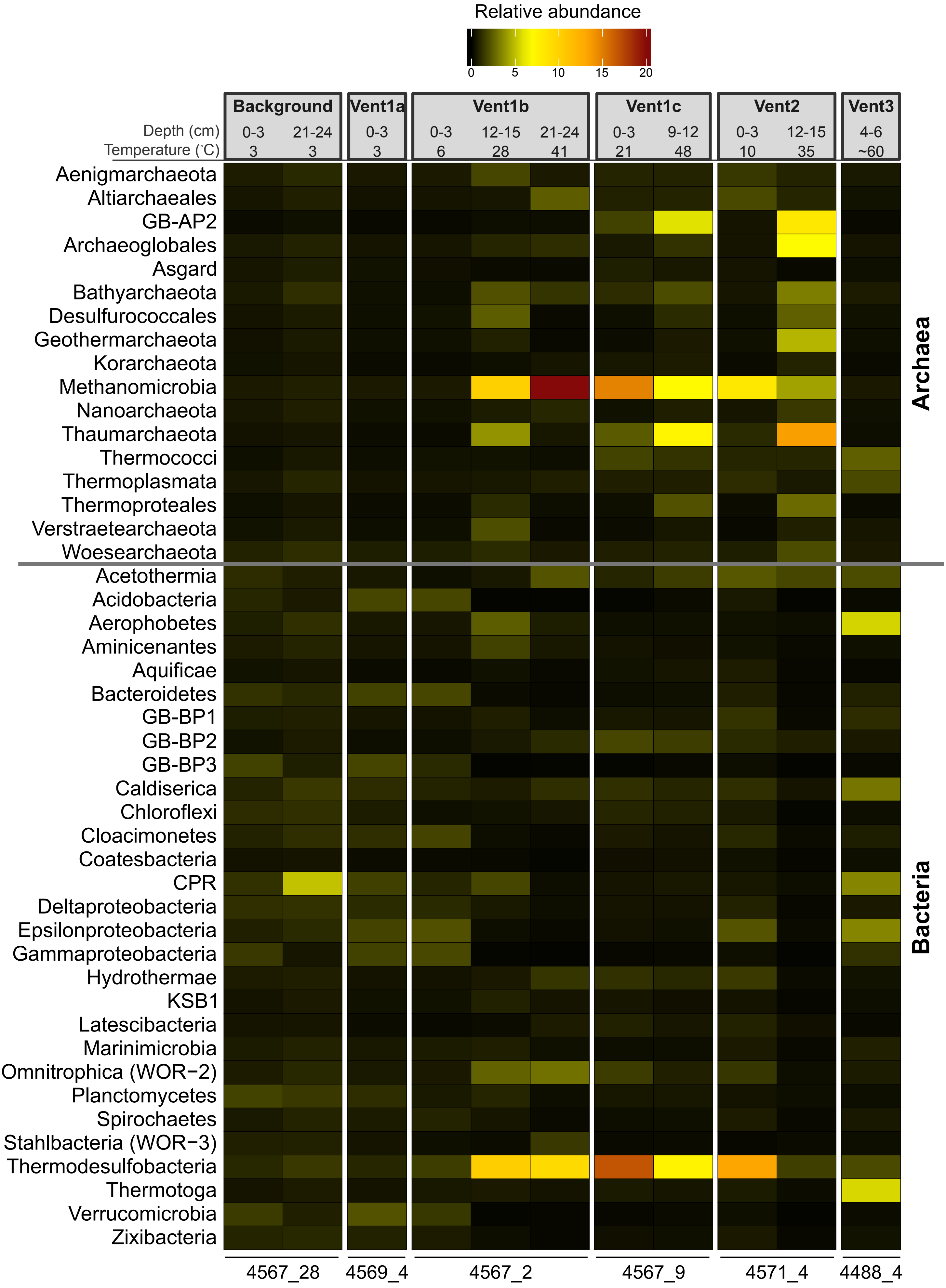

Supplementary Figure 5: Occurrence of major phylogenetic clusters across different GB sediments. Summarized count data of each individual genome belonging to a phylogenetic cluster (normalized by total contig length and library size) across different GB sediment locations, depth profiles and temperature regimes. Temperatures are averages for the 2 or 3 cm thick sediment layers from which DNA was isolated. Background samples: Cold GB samples without hydrothermal activity. Vent1-3: Hydrothermal sediment sampling locations, see also Fig. 1. Number codes at the bottom: The first four digits indicate the Alvin dive and the last digits identifies sediment cores, see also Supplementary Data 1 for further explanation.

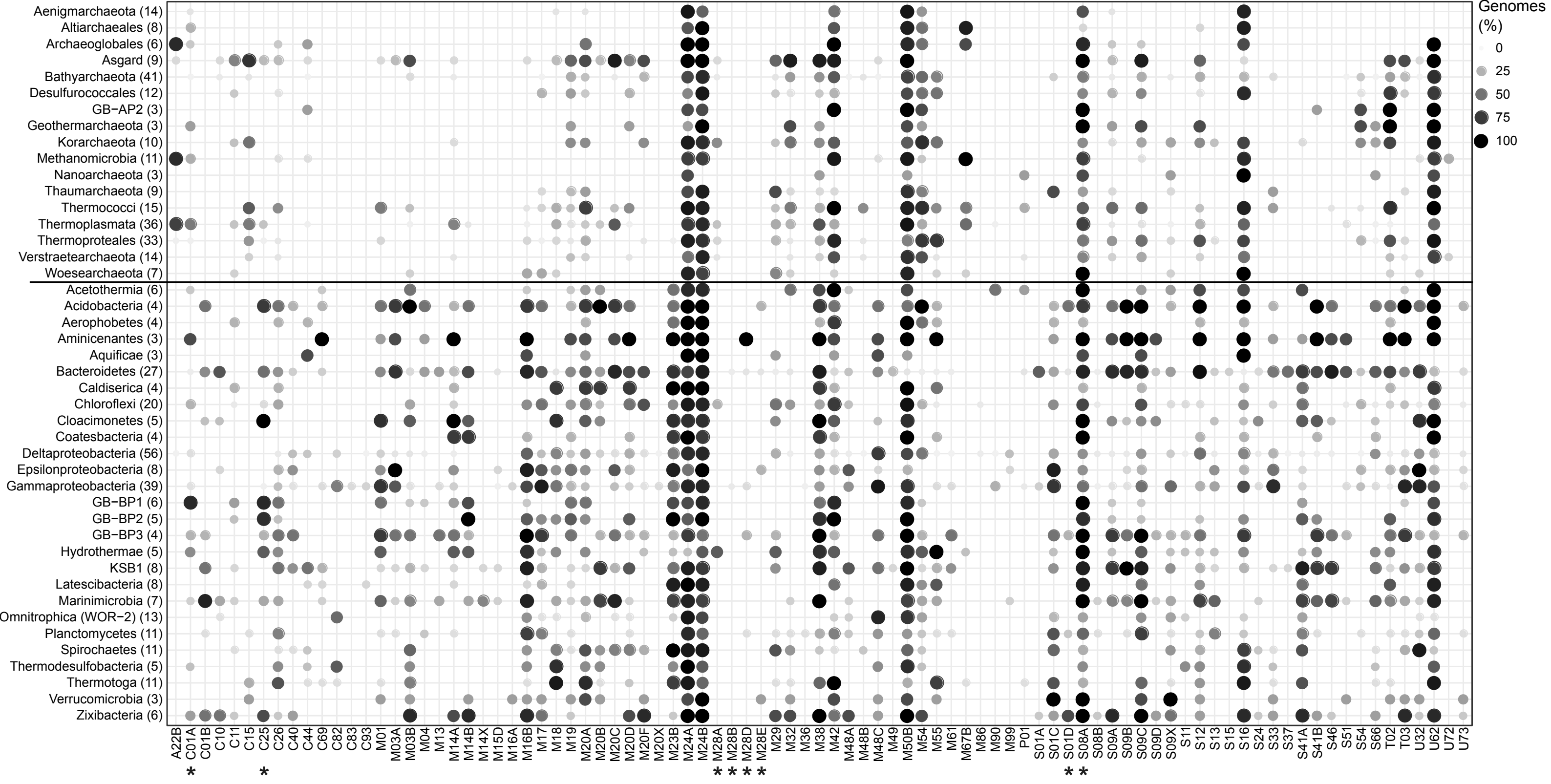

Supplementary Figure 6: Number of peptidases detected in GB genomes. Percentage of peptidases (Aspartic (A), Cysteine (C), Metallo (M), Mixed (P), Serine (S) peptidases) encoded in each GB genome (summarized for each phylogenetic cluster). Brackets: total number of genomes encoded in each phylogenetic cluster. Asterisk: Peptidase with potential secretion signal (see also Supplementary Data 9).

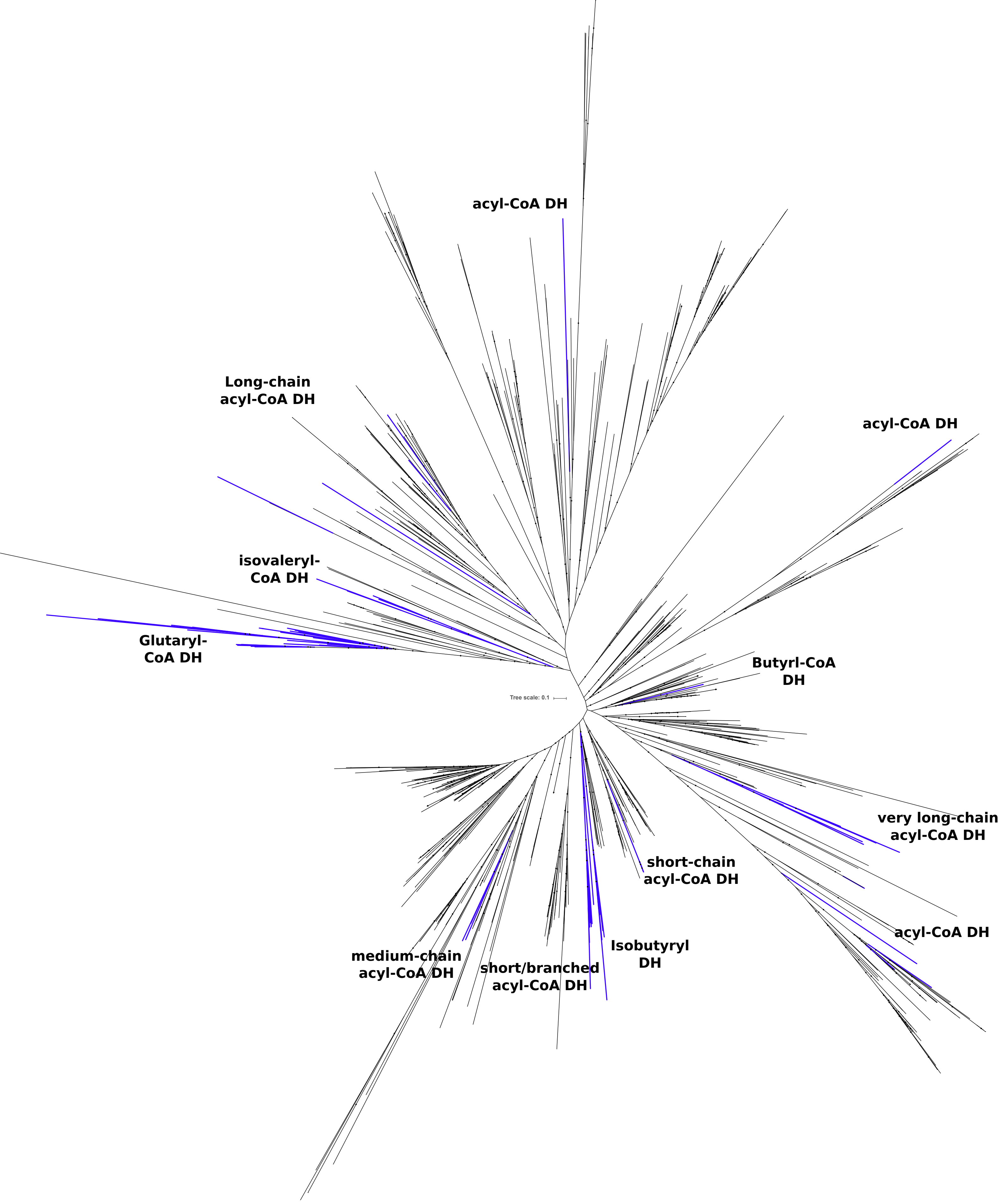

Supplementary Figure 7: Maximum likelihood phylogenetic tree of acyl-CoA dehydrogenases detected in GB genomes. Blue branches: Reference acyl-CoA dehydrogenase proteins. Black branches: Acyl-CoA dehydrogenases (DHs) detected in GB genomes. Black circles: Bootstrap support ≥ 70% (number of bootstraps determined using the extended majority-rule consensus tree criterion). RaxML was run as raxmlHPC-PTHREADS-AVX -f a -m PROTGAMMAAUTO -N autoMRE. The tree file is available in Supplementary Data 12.

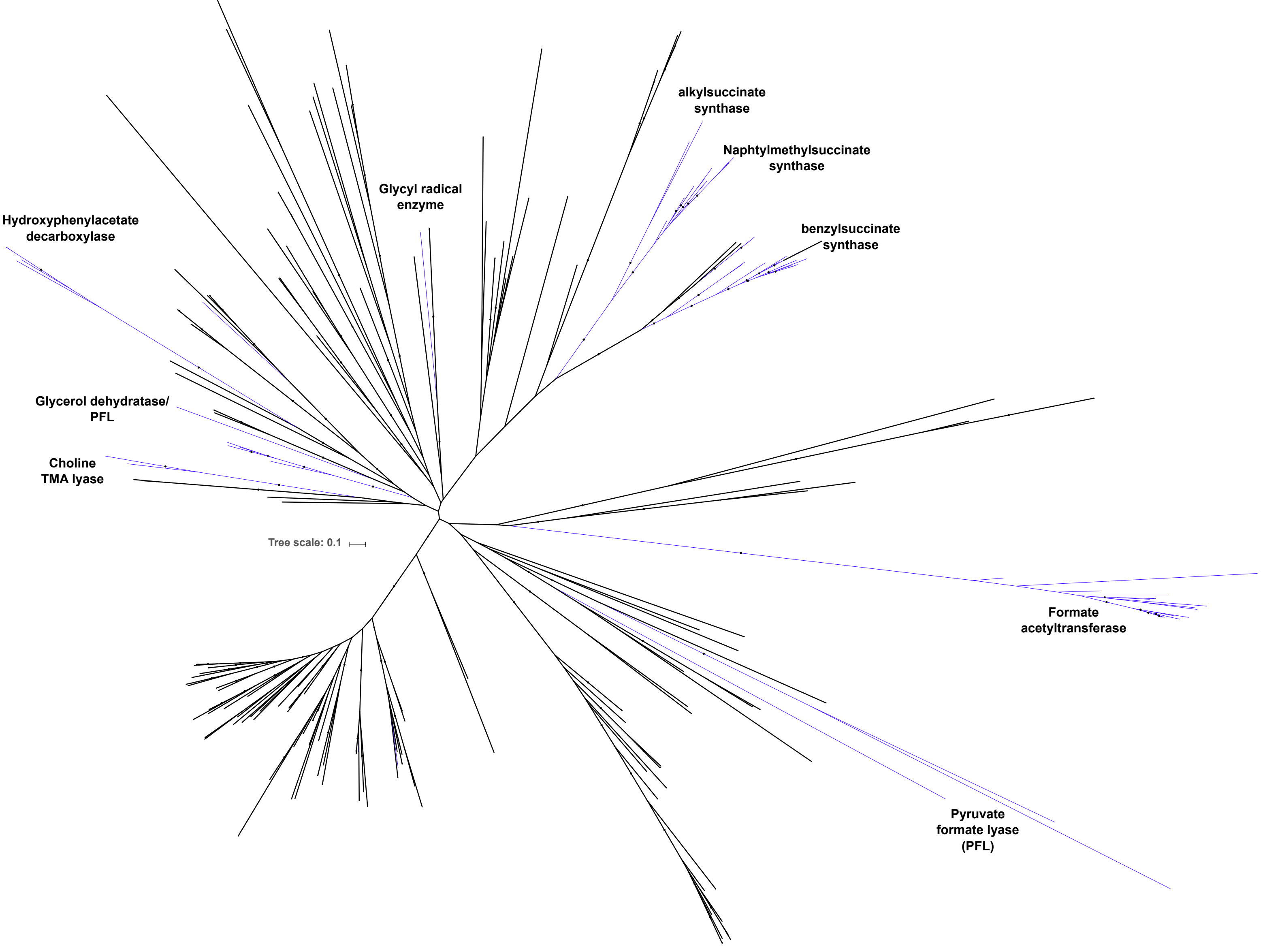

Supplementary Figure 8: Maximum likelihood phylogenetic tree of glycyl radical enzymes detected in GB MAGs. Blue branches: Reference glycyl radical enzymes. Black branches: Glycyl radical enzymes detected in GB MAGs (identified as *pflA*, *assA*, *bssA*, *hbsA*, *masD*, *nmsA* in KEGG or a custom blast search, see also Supplementary Table S9). Black circle: Bootstrap support ≥ 70 (number of bootstraps determined using the extended majority-rule consensus tree criterion). RaxML was run as raxmlHPC-PTHREADS-AVX -T 20 -f a -m PROTGAMMAAUTO -N autoMRE. The tree file is available in Supplementary Data 13.

Supplementary References

1. McKay, L. *et al.* Thermal and geochemical influences on microbial biogeography in the hydrothermal sediments of Guaymas Basin, Gulf of California. *Environ. Microbiol. Rep.* **8,** 150–161 (2016).
