# Supplementary material for: Expansive microbial metabolic versatility and biodiversity in dynamic Guaymas Basin hydrothermal sediments
Source: Nat Commun. 2018 Nov 27;9:4999. doi: 10.1038/s41467-018-07418-0 (PMC6258724; doi:10.1038/s41467-018-07418-0)
Supplement: Supplementary file 17 — Peer Review File [file 41467_2018_7418_MOESM17_ESM.pdf]

Reviewers' Comments:

Reviewer #1:
Remarks to the Author:
This manuscript by Dombrowski et al. describes genomes assembled from metagenomic sequencing of hydrothermal sediment from Guaymas Basin. From 11 samples from 6 different sediment cores, they assembled 551 genomes from 56 phyla, including a number of novel lineages. There are many similarities between this manuscript and an earlier paper from this group (Dombrowski et al, Microbiome, 2017), but the increased number of samples presented here allowed for interesting comparison between hydrothermal sediment and non-hydrothermal controls and between surface and deeper sediment, as well as additional insight into the genetic potential for anaerobic alkane oxidation. The paper is very well organized, reasonably well written, and their claims adequately supported by their data, including the very clearly presented supplemental figures and tables.

Some minor comments:

Ln 20: Protein-based seems like the wrong word unless you're actually doing proteomics

Ln: 221: Syntrophoarchaea, not Syntropharchaea

Ln 225: Cellvibrionales, not Cellovibrionales

Ln 239-240: "capable of short-chain alkane oxidation" is an over statement. It's possible, but you definitely haven't shown that yet.

Ln 350-57: How much do you think cell numbers change down core and between hydrothermal and background sediments? You say that you had trouble extracting DNA from the deep, hot sediments- does this imply you think the DNA was there but extraction yields were low for some reason, or that there were just fewer cells? Does this affect any of your discussions of diversity? The papers you're comparing to were measuring diversity differently- is there a reason you'd expect to see higher diversity based on this method?

Figure 6: For some reason this figure is really confusing me, though it seems like it shouldn't be that complicated. Can you clarify what the number and size and color of the circle mean and make all of the circles dark enough to actually see? For example, I don't understand why a 2 within the same sample is different colors and sizes for nirK and nirS. Why isn't the total number of genomes the same?

Reviewer #2:
Remarks to the Author:
Dombrowski et al present a comprehensive analysis of microbial metagenome-assembled genomes (MAGs) from a geochemically unique marine sediment environment, the hydrothermal and organic-enriched sediments of Guaymas Basin. The analysis focuses on diversity and functional gene content in hydrothermal-influenced sediments and compares these to patterns in 'background' non-hydrothermal samples, highlighting an enrichment in Archaeal diversity in higher temperature samples. The data are expansive and contain a number of MAGs whose nucleotide divergence and phylogenetic positioning suggest novel lineages, including five new phyla, although the latter are sparsely described (see below). Further, the results suggest previously unrecognized metabolic roles for certain major lineages of microbes, and also identify differences in the distributions of key genes of

hydrocarbon, C1, nitrogen, and sulfur metabolism, thereby expanding our understanding of how microbes may be organized into ecological niches.

What I like about this paper:

- The informatics analysis is meticulous and robust, and the results clearly presented. The figures, while complex (notably 3,4, and 6), distill diverse punchlines into clear composite images, with high information content that will likely be mined for hypotheses about specific taxa. The analysis undoubtedly took a lot of forethought and work - synthesizing such complex data into a set of poignant findings is not trivial.

- The paper continues an exciting tradition of using deep-sequencing to further the (seemingly endless) recovery of novel microbial lineages. Other recent studies have revealed similarly deep and underexplored pools of microbial diversity, such that we are at risk of taking these discoveries for granted. Here, five new candidate microbial phyla are described - this is a significant addition to our understanding of the tree of life. Moreover, the paper does a nice job of making a strong case for the unique environment of Guaymas (and perhaps deep-sea sediments in general) as models for probing the breadth of microbial diversification. I anticipate this work will be highly cited and, moreover, mined for patterns (or genomic templates) that can be the starting point for more comprehensive analyses of individual taxa.

- In addition to reporting novel lineages, the study highlights intriguing functional patterns in more extensively studied microbial groups. Again, given the wealth of information presented here at the community level, we are at risk of taking such taxon-specific findings for granted. However, some of these findings, including a putative role for methane metabolism in crenarchaeota, could likely stand alone as the basis for separate papers (with additional supporting analyses).

Concerns or questions:

- Five new phyla underdescribed. I was disappointed that the five new phyla are given scant attention beyond a discussion of their genomic divergence and phylogenetic placement. These taxa are included in Figures 2 and 4, but there is no discussion of their genomic features and presumed functional roles. Also, upon closer reading, it is unclear (notably from lines 116-112) what divergence criteria were actually used to identify these as new phyla. Based on Figure 2, one could argue that it looks like GB-AP1 and 2 are members of the Korarchaeota (which is misspelled on the tree btw). Based on comparable branch-length separation, why wouldn't the long branch extending out of the Chloroflexi clade also be considered a new phylum? The classification and description of these new phyla, if designated as such, require more attention in the text (particularly given their mention in the Abstract).

- Elevated archaeal richness in hydrothermal environments. It would be useful to more clearly emphasize why this result is particularly noteworthy (if indeed so). Given the historic association of Archaea with high temperature environments, this finding will likely not be surprising to readers, and they may question why it is a focal point of the Abstract and Discussion. You might consider downplaying this finding to bring more attention to the more novel components of the study (newly discovered lineages, functional patterns, etc).

- Sample numbers are low, thereby preventing strong conclusions about environment-diversity patterns. This is particularly true for the ‘background’ data, which come from a single core. Unfortunately, low replication is a consistent limitation of deep-sea work (it’s hard to get lots of samples). In my opinion, the strength of this paper is in its genomic exploration across the spectrum

of interesting microbes from this unique habitat - this should be highlighted as the primary focus. The results regarding the linkages between environmental variables (e.g., background vs hydrothermal vs oily sediments) and diversity patterns and functional redundancy are less compelling, because of the low sample number. However, I think there needs to be room for studies like this where logistics constrain the sampling below what's optimal, as long as the caveats are openly discussed. I therefore think the discussion of environmental drivers can remain in the paper, but it should be made clear that the patterns, while interesting, are based on a small number of samples, and follow-up studies are required to more rigorously link the genetic patterns to environmental determinants. The authors make this point at the end of the section about temperature and community assembly (lines 162-164), but it also applies to the later comparisons of gene content/functional redundancy between habitats, and should therefore be acknowledged more broadly in the discussion. Along these lines, the authors should identify and reword certain overly strong statements, such as "temperature drives community assembly." (Line 138). I don't doubt this is true, but the analysis doesn't actually test this empirically.

- The title is potentially overstated. "Extensive metabolic versatility and redundancy…" compared to what? A systematic and quantitative comparison across different habitats would be necessary to really know if the diversity patterns at Guaymas are exceptional.

- Following on the comment immediately above, the conclusions regarding 'functional redundancy' are also hard to interpret, namely as it isn't clear what the expectation is here. One could (reasonably?) argue that the presence of similar functions in diverse co-occurring taxa is an intrinsic property of microbial ecosystems. I.e., outside of engineered systems, certain low-complexity habitats (e.g., acidic springs), and certain other unique examples (e.g., anammox bacteria in OMZs), is it relatively rare that a major metabolic process is constrained to a single taxonomic unit? (Yes, I think) However, quantitatively comparing redundancy levels across habitats is not trivial (although this would be interesting), and isn't done here either. So it's again hard to determine if the pattern (redundancy) seen at Guaymas is exceptional. If 'functional redundancy' remains a theme of the paper, the text would benefit from establishing some expectations for when this is likely (or not likely) to occur. [also, it might be good to include a definition in the Intro of what this term actually means; this comes later, albeit indirectly, in the Discussion at line 390, but could be addressed earlier]

- The text is long (roughly 5000 words, not including the Methods). I understand why - there is a lot of great information here. However, I suspect the paper could be distilled to focus more concisely on the major punchlines.

Minor:

-lines 41-46: Awkward sentence structuring. Indeed, the Intro as a whole doesn't read quite as smoothly as other parts of the text. I suggest some minor editing here.

-line 84: It is unclear what you mean by "broadest spatial". Spanning the most habitat types? The largest distance between samples? Providing specifics about prior metagenomic analyses of hydrothermal sediments would help (e.g., Did prior work only focus on a single sample type? A single site? Much fewer reads? etc)

-line 555: How did you decide on the evolutionary model to use for your ML analysis?

Summary:

This is a valuable addition to the microbial diversity literature. The findings significantly expand our

knowledge of the diversity of major lineages, highlight previously unrecognized functional potential in certain major groups, and provide a starting point for studies targeting individual taxa and for deeper explorations of the environmental drivers of diversity in deep-sea sediments. Although the genomic analysis is meticulous and robust, the conclusions about environment-diversity linkages and functional redundancy are less compelling. The overall impact of the study could potentially be elevated by focusing more intently on the novel microbial groups and by more clearly establishing the rationale/expectations for why certain findings (e.g., redundancy, elevated archaeal diversity) are noteworthy.

Reviewer #3:
Remarks to the Author:
The manuscript by Dombrowski et al. presents the study of hydrothermal sediment samples from the Guaymas Basin, covering a wide range of physicochemical conditions. Microbial communities of the 11 analyzed samples have been characterized by means of genome binning from metagenomes. A total of 550 MAGs have recovered and their phylogenies and metabolic capabilities analyzed.
The subject is relevant, the paper is well written, the data have high quality, the analyses are sound and thorough, and the results are very interesting. However, this reviewer has two main concerns:
1. The novelty of the results compared to what was previously known about Guaymas basin hydrothermal sediments.
2. To which extent bins represent their respective communities.
Discussion of these points is scattered in the manuscript, but they should be made clearer.
Regarding the novelty, the authors should make clear whether the study supports or contradicts previous results of Guaymas Basin sediments. If the description of new candidate phyla is one of the main results of the paper, then they should be described in more detail.
Regarding the second point, all the study is based on the assumption that bins represent the community and therefore is of paramount relevance to make this point clear. The sentence in line 140-142 refers to an essential part of the study. Figure S4 (or another figure illustrating this point) should be moved to the main text and explained and discussed further. Indeed, according to Figure S4 the most abundant RPS3 in each metagenome have not been binned. Besides, according to table S4, not all bins contain RPS3. Finally, some metagenomes (Guaymas 11) are very poorly binned (18%).
A clear statement on how much metagenome is binned should be included in the text and in Table S2 (now is in Table S6). The phylogenetic diversity retrieved by binning could be compared, for instance, with that was obtained by contig annotation (or 16S read retrieval, or another approach) as a kind of control to check whether bins represent their corresponding metagenomes. Some statistical support should be provided to support the statements in lines 336-346 (for instance, regarding Table S10).
The authors surely have enough data to convince the readers that the MAGs are good representation of their communities, but these data must be shown in a clear way.

OTHER GENERAL COMMENTS
I appreciate the difficulty of taking the samples and their extraordinary value and understand that probably there is no more sample left, but it would be very relevant to know the abundance of cells in each sample (DAPI counts, FISH, etc.)
Maybe the authors would like to mention that functional redundancy is also observed in the human and the marine microbiomes (Structure, function and diversity of the healthy human microbiome. 2012. Nature 486S; Unagawa et al., Structure and function of the global ocean microbiome. 2015. Science 348)
Can something be said about the instraspecific diversity of the genomes or whether different "ecotypes" were found in different samples? When bins are present in different samples, are they identical? How do they change? Contig recruitment of the different bins in the analyzed metagenomes

could provide information on this point.
The analyses are very detailed and complete. However, going back and forth from the text to the tables difficults the smooth reading of the manuscript. The presentation of the results could improve by showing (i) a PCA graph with the physico-chemical characteristics of the samples and (ii) how different microbial groups (presence/absence or abundance of MAGs) are related to these environmental variables (CCA graph or some other graphical display, depending on the type of analysis).

SPECIFIC COMMENTS
Line 97: please use always the same numeration for cores (e.g 4569_2 instead of #2)
Line 107: the quality of the bins could be discussed further. The authors could use the criteria provided in Bowers, et al. Minimum information about a single amplified genome (MISAG) and a metagenome-assembled genome (MIMAG) of bacteria and archaea. Nat Biotechnol, 2017. 35(8): p. 725-731.
Line 153: the oily sediment is also the hotest sediment. How can the effect of these two aspects be distinguished? (Besides, it should read 4488_9). Is there any explanation for the low proportion of metagenome assembled? Microdiversity?
Lines 226-232: extremely interesting but highly speculative.
Line 313: is the comma necessary?
Line 359: according to supplementary Table S1, the temperature in the different layers of dive 4569_9 varies from 21.3 to 48.5
Line 344: is the community in deeper/hotter sediments a subset of this seedbank?
Line 365: which is the utility of the binning approach for detecting changes in functional capacities across sites? Wouldn't it be better to annotate all the contigs?
Line 380: many genomes (MAGs) were not complete. Is it then possible that some genes of certain metabolic pathways were not binned together? Could they have a mosaic structure?
Line 373: please, define "metabolic plasticity".
Line 412: to state this, we would need to know whether all the members of the community are active simultaneously.
Line 493: it would be very useful for the users of the binning approach to know how the different binning tools performed and to which extent Das Tool improved their outputs.
Lines 505-on (relative abundance): the calculation of the abundance of each MAG provided should also include the values of % of identity and coverage thresholds used for the analyses. It has to be clear that the abundance data shown in table S5 are the abundances of the complete genomes, not the abundances of their genes (that could be shared with other organisms). It would be helpful to show some recruitment plots for specific bins (such as the ones obtained with Enveomics; Rodriguez-R LM, Konstantinidis KT. (2016) The enveomics collection: a toolbox for specialized analyses of microbial genomes and metagenomes. PeerJ Preprints 4:e1900v1 https://doi.org/10.7287/peerj.preprints.1900v1)
Lines 558-560: very clear explanation (something rather unusual in this kind of works).
Refs 18, 29, 39: title is capitalized. Please, correct.
Figure S4: since the discussion about Bacteria and Archaea are separated throughout the manuscript, it would be helpful to have two sets of graphs of RPS3 abundance, one for Archaea and one for Bacteria, or just a different color codes for them in the current graph.
Table S5 caption: "The order in the table reflects the order of the tree provided in Supplementary Figure 1". This must be a mistake since Suplementary Fig 1 shows "Temperature variability in GB sediments"

Response to reviewers:

Many thanks to all the reviewers for the detailed comments, we hope we have covered all concerns sufficiently and very much appreciate the time that went into writing such detailed reviews. Our answers to each raised comment are outlined below and each edit can be found in the marked-up word document of the manuscript (the line numbers below refer to this version).

Reviewer #1 (Remarks to the Author):

This manuscript by Dombrowski et al. describes genomes assembled from metagenomic sequencing of hydrothermal sediment from Guaymas Basin. From 11 samples from 6 different sediment cores, they assembled 551 genomes from 56 phyla, including a number of novel lineages. There are many similarities between this manuscript and an earlier paper from this group (Dombrowski et al, Microbiome, 2017), but the increased number of samples presented here allowed for interesting comparison between hydrothermal sediment and non-hydrothermal controls and between surface and deeper sediment, as well as additional insight into the genetic potential for anaerobic alkane oxidation. The paper is very well organized, reasonably well written, and their claims adequately supported by their data, including the very clearly presented supplemental figures and tables.

Thanks to the reviewer for the kind comments, we appreciate the input and hope that we could respond appropriately to all concerns.

Some minor comments:

1. Ln 20: Protein-based seems like the wrong word unless you're actually doing proteomics

We agree and have changed the text to 'Genome-based metabolic inferences' in Line 23.

2. Ln: 225: Syntrophoarchaea, not Syntropharchaea

This was corrected in Ln 231 to Syntrophoarchaea.

3. Ln 225: Cellvibrionales, not Cellovibrionales

This was corrected in Ln 235 to Cellvibrionales.

4. Ln 239-240: "capable of short-chain alkane oxidation" is an over statement. It's possible, but you definitely haven't shown that yet.

We agree with the reviewer that this should be described more carefully and accordingly changed the text in Ln 252 to 'potentially able to use short-chain alkanes'.

5. Ln 350-57: How much do you think cell numbers change down core and between hydrothermal and background sediments? You say that you had trouble extracting DNA from the deep, hot sediments- does this imply you think the DNA was there but extraction yields were low for some reason, or that there were just fewer cells? Does this affect any of your discussions of diversity? The papers you're comparing to were measuring diversity differently- is there a reason you'd expect to see higher diversity based on this method?

Cell counts are expected to decrease with depth (see also Figure 3 in Meyer et al. 2013, that describes cell counts/OTU numbers from comparable sites in GB), which would explain difficulties in extracting DNA from deeper sites. However, consistent with our diversity estimates the decrease in cell numbers does not necessarily correlate with a decrease in OTU numbers (at least at depths with cold to medium temperature). Therefore, we do not believe that DNA extraction was low due to technical difficulties and thus this issue should not affect our discussion in diversity. Finally, it is true that the cited papers use different methods for measuring diversity but we do not see a technical issue with our approach that would artificially increase the diversity we see. To account for the reviewers concern, we now added this part in the discussion, Lines 371-375, which now states 'Earlier work reported a decrease in cell

numbers with increasing depth that did not necessarily correlate with a decrease in OTU numbers25, potentially explaining our difficulties in isolating sufficient amounts of DNA but supporting our assumption that steep temperature gradients do not necessarily inhibit microbial diversity.'

1. Meyer, S. *et al.* Microbial habitat connectivity across spatial scales and hydrothermal temperature gradients at Guaymas Basin. *Front Microbiol* 4, (2013).

6. Figure 6: For some reason this figure is really confusing me, though it seems like it shouldn't be that complicated. Can you clarify what the number and size and color of the circle mean and make all of the circles dark enough to actually see? For example, I don't understand why a 2 within the same sample is different colors and sizes for nirK and nirS. Why isn't the total number of genomes the same?

For the given example the circle size corresponds to the number of genomes encoding nirK/total number of genomes at a certain site (so the percentage of the whole community encoding a certain gene). In contrast, the number in the circle refers to the number of phyla encoding for nirK at a certain site, which is why even if the numbers are the same the size of the circle can be different. To make this clearer, we added some extra description in the figure and clarified this in the legend.

Reviewer #2 (Remarks to the Author):

Dombrowski et al present a comprehensive analysis of microbial metagenome-assembled genomes (MAGs) from a geochemically unique marine sediment environment, the hydrothermal and organic-enriched sediments of Guaymas Basin. The analysis focuses on diversity and functional gene content in hydrothermal-influenced sediments and compares these to patterns in 'background' non-hydrothermal samples, highlighting an enrichment in Archaeal diversity in higher temperature samples. The data are expansive and contain a number of MAGs whose nucleotide divergence and phylogenetic positioning suggest novel lineages, including five new phyla, although the latter are sparsely described (see below). Further, the results suggest previously unrecognized metabolic roles for certain major lineages of microbes, and also identify differences in the distributions of key genes of hydrocarbon, C1, nitrogen, and sulfur metabolism, thereby expanding our understanding of how microbes may be organized into ecological niches.

What I like about this paper:

- The informatics analysis is meticulous and robust, and the results clearly presented. The figures, while complex (notably 3,4, and 6), distill diverse punchlines into clear composite images, with high information content that will likely be mined for hypotheses about specific taxa. The analysis undoubtedly took a lot of forethought and work - synthesizing such complex data into a set of poignant findings is not trivial.

We are glad to hear that our manuscript is easily understandable and we agree that it always challenging to write a comprehensive overview using metagenomic data.

- The paper continues an exciting tradition of using deep-sequencing to further the (seemingly endless) recovery of novel microbial lineages. Other recent studies have revealed similarly deep and underexplored pools of microbial diversity, such that we are at risk of taking these discoveries for granted. Here, five new candidate microbial phyla are described - this is a significant addition to our understanding of the tree of life. Moreover, the paper does a nice job of making a strong case for the unique environment of Guaymas (and perhaps deep-sea sediments in general) as models for probing the breadth of microbial diversification. I anticipate this work will be highly cited and, moreover, mined for patterns (or genomic templates) that can be the starting point for more comprehensive analyses of individual taxa.

Thanks for this comment, we agree that there is ample opportunity to discover new microbial lineages and are glad that our paper can continue adding new lineages to the tree of life.

- In addition to reporting novel lineages, the study highlights intriguing functional patterns in more extensively

studied microbial groups. Again, given the wealth of information presented here at the community level, we are at risk of taking such taxon-specific findings for granted. However, some of these findings, including a putative role for methane metabolism in crenarchaeota, could likely stand alone as the basis for separate papers (with additional supporting analyses).

We agree and since we believe that it is impossible to discuss all of this information in detail within a single paper we are currently preparing separate papers with regards to certain novel and interesting groups. In the interest of examining the broader community inhabiting Guaymas Basin sediments, we decided to include as much as possible in this study and thereby also provide the data to the research community early on.

Concerns or questions:

7. - Five new phyla under described. I was disappointed that the five new phyla are given scant attention beyond a discussion of their genomic divergence and phylogenetic placement. These taxa are included in Figures 2 and 4, but there is no discussion of their genomic features and presumed functional roles. Also, upon closer reading, it is unclear (notably from lines 116-112) what divergence criteria were actually used to identify these as new phyla. Based on Figure 2, one could argue that it looks like GB-AP1 and 2 are members of the Korarchaeota (which is misspelled on the tree btw). Based on comparable branch-length separation, why wouldn't the long branch extending out of the Chloroflexi clade also be considered a new phylum? The classification and description of these new phyla, if designated as such, require more attention in the text (particularly given their mention in the Abstract).

We are in the process of doing a closer examination of the new phyla presented here. These papers will provide a comprehensive examination of the metabolisms and phylogenies of these novel phyla and we fear that discussing them here in depth will dilute information too much. However, since the aim of this study was to give a comprehensive overview about the diversity present in Guaymas Basin sediments, we also did not want to exclude any taxa and mention them in the abstract to overall highlight the novel diversity that can be found within this sampling location. Additionally, while we do not discuss the new candidate phyla in detail in the results section, the interested reader can extract information on their genomic repertoire from table S9 (now Supplementary Data 10).

We agree that identifying new phyla can be difficult especially since at the moment there is no widely accepted definition of what constitutes a new phylum (i.e. when relying on branch-length such a placement is strongly depend on the model used for generating the tree). Additionally, we could only extract 16S rRNA sequences from one lineage (GB-BP1), therefore we cannot use this marker gene to place the remaining new phyla. For all these reasons, we decided to guide our phylum-definition on whole-genome based AAI inferences. We added additional information on this in the Methods, Lines 580-584 and explain this very briefly in the results section (Lines 125-127). To give an example: GB-AP1 shares on average ~44.5% AAI and GB-AP2 ~46% AAI with Korarchaeota. In contrast the AAI of genomes within the Korarchaeota is ~ 56%. Thus, we believed the identity is too low to assign these new lineages to the Korarchaoeta.

Thanks for noticing, Korarchaeota was corrected in Fig. 2

8. - Elevated archaeal richness in hydrothermal environments. It would be useful to more clearly emphasize why this result is particularly noteworthy (if indeed so). Given the historic association of Archaea with high temperature environments, this finding will likely not be surprising to readers, and they may question why it is a focal point of the Abstract and Discussion. You might consider downplaying this finding to bring more attention to the more novel components of the study (newly discovered lineages, functional patterns, etc).

Yes, historically archaea are associated with extreme environments. What was most surprising to us is that these are the first communities we have sequenced that allowed us to reconstruct more archaeal than bacterial genomes. However, we appreciate the idea of highlighting the novelty of greater archaeal diversity, and changed the both the title and abstract accordingly (i.e. Ln 20-23).

9. - Sample numbers are low, thereby preventing strong conclusions about environment-diversity patterns. This is particularly true for the 'background' data, which come from a single core. Unfortunately, low replication is a consistent limitation of deep-sea work (it's hard to get lots of samples). In my opinion, the strength of this paper

is in its genomic exploration across the spectrum of interesting microbes from this unique habitat - this should be highlighted as the primary focus. The results regarding the linkages between environmental variables (e.g., background vs hydrothermal vs oily sediments) and diversity patterns and functional redundancy are less compelling, because of the low sample number. However, I think there needs to be room for studies like this where logistics constrain the sampling below what's optimal, as long as the caveats are openly discussed. I therefore think the discussion of environmental drivers can remain in the paper, but it should be made clear that the patterns, while interesting, are based on a small number of samples, and follow-up studies are required to more rigorously link the genetic patterns to environmental determinants. The authors make this point at the end of the section about temperature and community assembly (lines 162-164), but it also applies to the later comparisons of gene content/functional redundancy between habitats, and should therefore be acknowledged more broadly in the discussion. Along these lines, the authors should identify and reword certain overly strong statements, such as "temperature drives community assembly." (Line 138). I don't doubt this is true, but the analysis doesn't actually test this empirically.

It is true that the low sample numbers (as well as for example the lack of functional activity inferences) are important caveats that should be highlighted in the manuscript. Accordingly, we now mention these potential issues in the discussion (lines 441-447) to state 'A limitation of the current study that complicates a definite description of the diversity patterns and functional redundancy present in Guaymas sediments is the low sample number and limited number of bins recovered from a subset of samples (i.e. 4567_28 and 4488_9); given the limitations of deep-sea sampling, different habitat and sediment types are represented unevenly. Activity-based analyses of large sample numbers, i.e. metatranscriptomics, would more rigorously link genetic patterns to their environmental determinants.'

Additionally, we reworded overly strong statements throughout the manuscript. For example, the section title in line 146 was rewritten more generally and now reads 'The influence of environmental parameters on community assembly in hydrothermal sediments'.

10. - The title is potentially overstated. "Extensive metabolic versatility and redundancy…" compared to what? A systematic and quantitative comparison across different habitats would be necessary to really know if the diversity patterns at Guaymas are exceptional.

We agree with the reviewer that the title could be more precise; accordingly we changed it to 'Expansive microbial metabolic versatility and biodiversity in dynamic Guaymas Basin hydrothermal sediments'.

11. - Following on the comment immediately above, the conclusions regarding 'functional redundancy' are also hard to interpret, namely as it isn't clear what the expectation is here. One could (reasonably?) argue that the presence of similar functions in diverse co-occurring taxa is an intrinsic property of microbial ecosystems. I.e., outside of engineered systems, certain low-complexity habitats (e.g., acidic springs), and certain other unique examples (e.g., anammox bacteria in OMZs), is it relatively rare that a major metabolic process is constrained to a single taxonomic unit? (Yes, I think) However, quantitatively comparing redundancy levels across habitats is not trivial (although this would be interesting), and isn't done here either. So it's again hard to determine if the pattern (redundancy) seen at Guaymas is exceptional. If 'functional redundancy' remains a theme of the paper, the text would benefit from establishing some expectations for when this is likely (or not likely) to occur. [also, it might be good to include a definition in the Intro of what this term actually means; this comes later, albeit indirectly, in the Discussion at line 390, but could be addressed earlier]

Upon reflection, we agree with the reviewers concern. Since redundancy is not quantified, we decided to remove this term in the new title. Overall, we changed the title to highlight more the microbial diversity we find in the sediments and toned down appropriate sentences throughout the manuscript (i.e. Line 29). We still keep the discussion of this concept at the end, where we added a definition of the term 'functional redundancy' (Lines 409-412) to state '…the metabolic repertoire shows a high degree of functional redundancy across different phyla, i.e. different taxa encode the same metabolic function and thus might substitute for one another. Therefore, even if community composition varies, metabolic function is predicted to be relatively stable.'.

12. - The text is long (roughly 5000 words, not including the Methods). I understand why - there is a lot of great information here. However, I suspect the paper could be distilled to focus more concisely on the major punchlines.

While it is true that the text is at the word limit for Nature Communications, we believe that the wealth of information provided by the genomic data justifies the length of the manuscript, especially considering that shortening the text could easily result in a harder to digest manuscript.

Minor:

13. -lines 41-46: Awkward sentence structuring. Indeed, the Intro as a whole doesn't read quite as smoothly as other parts of the text. I suggest some minor editing here.

Agreed, this sentence and the introduction have been adjusted to read more clearly. The sentence now reads 'These compounds migrate to the sediment surface with rising vent fluids, where they fuel hydrocarbon-degrading microbial communities. Among all hydrothermally generated hydrocarbons, methane has received considerable interest as greenhouse gas shaping global climate. Porewater methane reaches millimolar concentrations while ethane ranges from 40-100 µM. Also present in these sediments are propane, n-butane and propane, which accumulate at lower concentrations compared to methane. Altogether, these hydrocarbons represent lucrative carbon sources for the resident microbial community.'

14. -line 84: It is unclear what you mean by "broadest spatial". Spanning the most habitat types? The largest distance between samples? Providing specifics about prior metagenomic analyses of hydrothermal sediments would help (e.g., Did prior work only focus on a single sample type? A single site? Much fewer reads? etc)

This sentence was removed in the revised manuscript, also considering that as time passes there likely will be further studies on Guaymas.

15. -line 555: How did you decide on the evolutionary model to use for your ML analysis?

We used the GTRGAMMA over the GTRCAT model, since the latter is usually not recommended for smaller tree (as is the case in our study).

Summary:

This is a valuable addition to the microbial diversity literature. The findings significantly expand our knowledge of the diversity of major lineages, highlight previously unrecognized functional potential in certain major groups, and provide a starting point for studies targeting individual taxa and for deeper explorations of the environmental drivers of diversity in deep-sea sediments. Although the genomic analysis is meticulous and robust, the conclusions about environment-diversity linkages and functional redundancy are less compelling. The overall impact of the study could potentially be elevated by focusing more intently on the novel microbial groups and by more clearly establishing the rationale/expectations for why certain findings (e.g., redundancy, elevated archaeal diversity) are noteworthy.

Reviewer #3 (Remarks to the Author):

The manuscript by Dombrowski et al. presents the study of hydrothermal sediment samples from the Guaymas Basin, covering a wide range of physicochemical conditions. Microbial communities of the 11 analyzed samples have been characterized by means of genome binning from metagenomes. A total of 550 MAGs have recovered and their phylogenies and metabolic capabilities analyzed.
The subject is relevant, the paper is well written, the data have high quality, the analyses are sound and thorough, and the results are very interesting. However, this reviewer has two main concerns:

16.
(a) The novelty of the results compared to what was previously known about Guaymas basin hydrothermal sediments. Regarding the novelty, the authors should make clear whether the study supports or contradicts previous results of Guaymas Basin sediments. If the description of new candidate phyla is one of the main results of the paper, then they should be described in more detail.

The results of this study are consistent with findings from earlier work but the higher number of spatial data (especially the availability of background samples, which were not available during the course of our earlier study) and 5x higher number of recovered genomes allowed as to draw better conclusions about the site as a whole. Accordingly, we specifically highlighted the presence of background samples in the abstract, as well as individual novel findings (such as the presence of putative alkane utilization pathways in ANME-1) in the manuscript. To extend this, we added a short mention of these features in the beginning of the discussion section (Lines 361-364), where we state 'Compared to earlier work on Guaymas Basin sediments, the higher sampling number and inclusion of background samples allowed to better describe the enhanced diversity present in these sediments as well as shed light on the drivers of community assembly.'.

As mentioned in the response to Reviewer 2 (see also point 7), we intend to describe the candidate phyla in more detail, however, since the scope of this work is to provide an overview of Guaymas Basin as a whole, we decided to describe these phyla in separate papers, which are in writing right now.

(b). To which extent bins represent their respective communities. All the study is based on the assumption that bins represent the community and therefore is of paramount relevance to make this point clear. The sentence in line 140-142 refers to an essential part of the study. Figure S4 (or another figure illustrating this point) should be moved to the main text and explained and discussed further. Indeed, according to Figure S4 the most abundant RPS3 in each metagenome have not been binned. Besides, according to table S4, not all bins contain RPS3. Finally, some metagenomes (Guaymas 11) are very poorly binned (18%).
A clear statement on how much metagenome is binned should be included in the text and in Table S2 (now is in Table S6). The phylogenetic diversity retrieved by binning could be compared, for instance, with that was obtained by contig annotation (or 16S read retrieval, or another approach) as a kind of control to check whether bins represent their corresponding metagenomes. Some statistical support should be provided to support the statements in lines 336-346 (for instance, regarding Table S10). The authors surely have enough data to convince the readers that the MAGs are good representation of their communities, but these data must be shown in a clear way.

Thanks to the reviewer for this detailed comment, we decided against moving Figure S4 into the main text, but added the distribution of archaea vs bacteria for the binned contigs/site. We also expanded the method section to discuss the assembly statistics further (Lines 538-540) and the percentage of binned reads was added to Supplementary Table S2 (now Supplementary Data 2). However, we do agree that for samples for which less bins were recovered (i.e. G1, G2 and G17), there is the risk that certain taxa are missed. Therefore, we mentioned this in the caveats considered in the end of the discussion (Lines 441-447).

It is true that in some, but not all cases, the most abundant RPS3 was not binned (numbers were now added in Line 557). However, since the metabolic genes determined using the binned genomes was comparable to patterns determined using the whole assembly (see also Lines 351-353), we believe that the bins represent the community pretty well at least in terms of their functional potential.

Finally, we would love to provide statistical support for the MAGs representing the community as a whole, however, considering the fact that the strong environmental variabilities will likely also result in shifts in community patterns, repeated sampling would be needed to draw stronger conclusions. Many environmental determinants become clear only post-cruise, when thermal and geochemical analyses of the samples are completed and the results compiled, and the sampling gaps and statistical problem areas are becoming fully apparent. We are returning to Guaymas Basin on scheduled cruises in 2018 and 2019, and will compensate for past sampling gaps; in multiple iterations it should be possible to statistically "cover" the microbial diversity of Guaymas Basin. As a caveat, this sample set and survey has not even touched the chimneys… more work needs to be done.

OTHER GENERAL COMMENTS
I appreciate the difficulty of taking the samples and their extraordinary value and understand that probably there is no more sample left, but it would be very relevant to know the abundance of cells in each sample (DAPI counts, FISH, etc.)

Yes, we are very interested in that information as well. Unfortunately, nothing is left of these particular samples. Preliminary cell counts of Guaymas hydrothermal sediments obtained during the same

cruises in 2008/2009 indicated very high cell densities near 10exp11 cells/ml in the upper 3-5 cm sediments, followed by rapidly declining numbers immediately below. However, we are collecting new samples this fall and plan to do systematic cell counts for a follow up study. However, we now discuss work from an earlier study describing cell counts and OTU numbers from nearby sites (Ln 371-376), where we state 'Earlier work reported a decrease in cell numbers with increasing depth that did not necessarily correlate with a decrease in OTU numbers, potentially explaining our difficulties in isolating sufficient amounts of DNA but supporting our assumption that steep temperature gradients do not necessarily inhibit microbial diversity'.

17.Maybe the authors would like to mention that functional redundancy is also observed in the human and the marine microbiomes (Structure, function and diversity of the healthy human microbiome. 2012. Nature 486S; Unagawa et al., Structure and function of the global ocean microbiome. 2015. Science 348)

Thanks for this suggestion, the references were now integrated in the manuscript (Lines 413-414).

18.Can something be said about the instraspecific diversity of the genomes or whether different "ecotypes" were found in different samples? When bins are present in different samples, are they identical? How do they change? Contig recruitment of the different bins in the analyzed metagenomes could provide information on this point.

While the authors have not looked in detail at intra-specific diversity, the AAI comparisons provide evidence that most genomes are indeed not the same (i.e. no genomes share 100% AAI but 59 genomes share an AAI >99% with other GB genomes, i.e. across the Gammaproteobacteria as seen in SI Table 5, now Supplementary Data 6). However, there does not seem to be an obvious trend based on sample location. We agree that contig recruitment would give more insights into strain variability, however, this is not really the scope of this study. To address some of the reviewers concerns, we now mention in more details on the AAI analyses in the methods section (Lines 580-584).

19. The analyses are very detailed and complete. However, going back and forth from the text to the tables difficults the smooth reading of the manuscript. The presentation of the results could improve by showing (i) a PCA graph with the physico-chemical characteristics of the samples and (ii) how different microbial groups (presence/absence or abundance of MAGs) are related to these environmental variables (CCA graph or some other graphical display, depending on the type of analysis).

Thanks for the suggestion, we agree that this would be an interesting analysis, however, we feel that we are lacking enough data points, both in terms of number of sites studied as well as environmental parameters measured, to do a statistically sound correlation analysis. However, we will build on this dataset and add more samples, environmental settings as well as chemical data as we are returning to Guaymas Basin over the years.

SPECIFIC COMMENTS
20. Line 97: please use always the same numeration for cores (e.g 4569_2 instead of #2)

To make the text more consistent, we removed the hash in Ln 103.

21. Line 107: the quality of the bins could be discussed further. The authors could use the criteria provided in Bowers, et al. Minimum information about a single amplified genome (MISAG) and a metagenome-assembled genome (MIMAG) of bacteria and archaea. Nat Biotechnol, 2017. 35(8): p. 725-731.

All genomes could be defined as medium-quality bins according to Bowers et al., 2017 (mainly due to the difficulty in assembling the 16S and higher number of contigs/bin). This information was now added to the text in Ln 114.

22. Line 153: the oily sediment is also the hottest sediment. How can the effect of these two aspects be distinguished? (Besides, it should read 4488_9). Is there any explanation for the low proportion of metagenome assembled? Microdiversity?

The text was changed to 4488_9.

As discussed in Ln 171-173 we believe that a larger sampling size of oily sediments would be needed to disentangle the relative contribution of temperature versus hydrocarbon content.

Unfortunately, we do not know what explains the difficulties in assembling some of the metagenomes, based on a quick phylogenetic analysis of unbinned RPS3 sequences, these sequences are distributed across the tree of life and thus do not hint towards a microdiversity issue.

23. Lines 226-232: extremely interesting but highly speculative.

We agree that this comment is speculative and tried to down-tone the sentence but since the phylogenetic statement is consistent with findings from Syntrophoarchaeum, we still would like to include it.

24. Line 313: is the comma necessary?

No, the comma was removed (now Ln 325)

25. Line 359: according to supplementary Table S1, the temperature in the different layers of dive 4569_9 varies from 21.3 to 48.5

This value refers to Ref 17 in the manuscript; temperature loggers inserted into the sediment at different depths monitored the temperature variance over a longer time window [8 days]. The temperatures in Supplementary Table S1 (now Supplementary Data 1) represent different sediment layers at the time when the thermal profile was checked using the Alvin heat flow probe just before sampling. The text now mentions long-term thermal logging explicitly (Line 377).

26. Line 344: is the community in deeper/hotter sediments a subset of this seedbank?

Yes, as we mention in Ln 380-382, we would assume that the deeper sediments select for a subset of the shallow community.

27. Line 365: which is the utility of the binning approach for detecting changes in functional capacities across sites? Wouldn't it be better to annotate all the contigs?

The benefit of annotating contigs that were binned is that it easier to link them with a potential organism and put them in context with other genes that are binned into the same genome. While unbinned contigs can be taxonomically assigned, incomplete databases for metabolic genes as well as horizontal gene transfer can make the interpretation of data very difficult. However, we used annotations from all contigs as means to confirm our assumptions (see also Ln 352-357) .

28. Line 380: many genomes (MAGs) were not complete. Is it then possible that some genes of certain metabolic pathways were not binned together? Could they have a mosaic structure?

While we *cannot exclude this possibility, we believe this is unlikely. For most lineages, we draw our conclusions not only from one MAG but from several MAGs belonging to the same phylogenetic cluster, which lowers the chance of missing a gene due to incompleteness. Additionally, several other studies argue for the existence of mosaic genomes/ metabolic trait-offs (Refs 43,44 in the manuscript).*

29. Line 373: please, define "metabolic plasticity".

This definition was now added 'metabolic plasticity, i.e. switching metabolic processes in response to changes in environmental conditions.' (Line 391)

20. Line 412: to state this, we would need to know whether all the members of the community are active simultaneously.

To better highlight caveats of this study, we added a short mentioning of potential issues at the end of the discussion (Lines 441-447).

21. Line 493: it would be very useful for the users of the binning approach to know how the different binning tools performed and to which extent Das Tool improved their outputs.

We tested DAS Tool initially for a subset of samples and did not do rigorous testing for the full dataset as we did not intend to do a complete benchmarking. From these initial analyses we noticed that DAS Tool allowed us to (a) reduce megabins (i.e. bins consisting of more than 1 genome) and increase overall completeness and decrease overall contamination. Below is an example of the statistics for 4571_4.

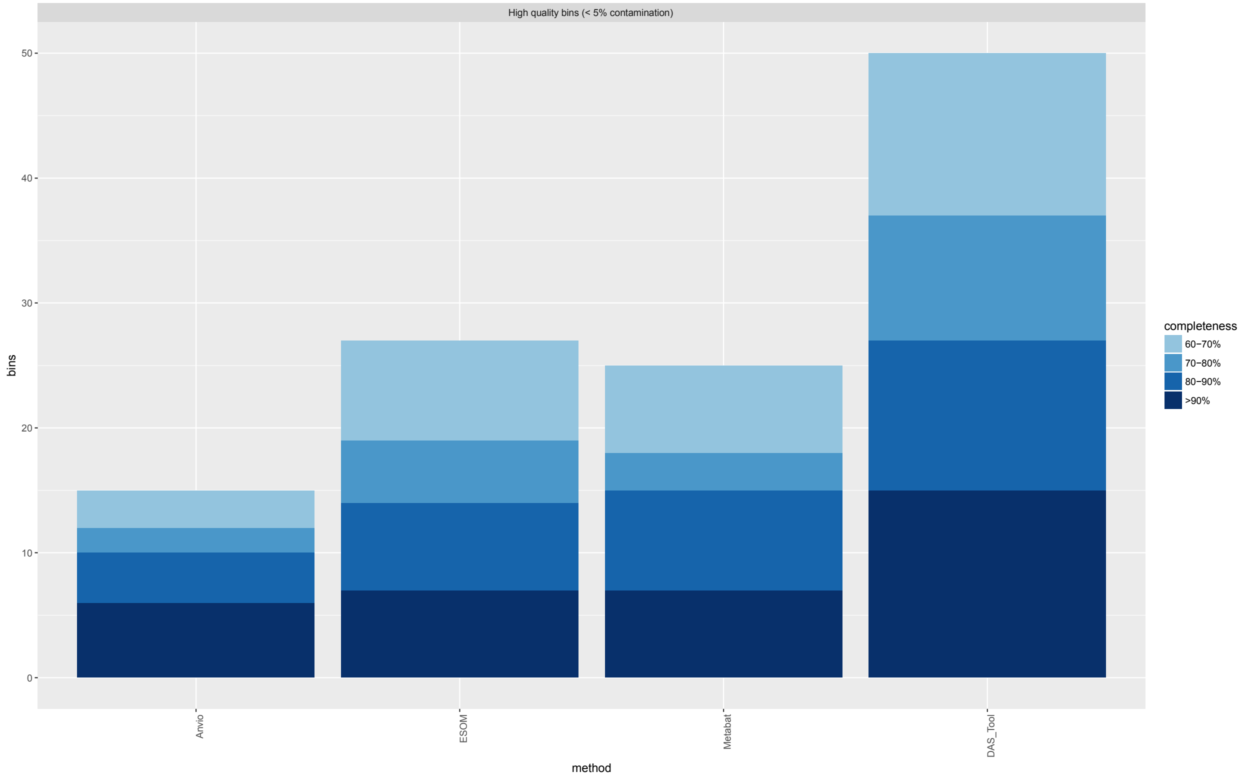

22. Lines 505-on (relative abundance): the calculation of the abundance of each MAG provided should also include the values of % of identity and coverage thresholds used for the analyses. It has to be clear that the abundance data shown in table S5 are the abundances of the complete genomes, not the abundances of their genes (that could be shared with other organisms). It would be helpful to show some recruitment plots for specific bins (such as the ones obtained with Enveomics; Rodriguez-R LM, Konstantinidis KT. (2016) The enveomics collection: a toolbox for specialized analyses of microbial genomes and metagenomes. PeerJ Preprints 4:e1900v1 https://doi.org/10.7287/peerj.preprints.1900v1)

Thanks for the suggestion, accordingly, we added an additional sentence to the caption of Table S3 (now Supplementary Data 3) to explain better what "relative abundance" refers to. As mentioned in the methods section, we used BWA with the default settings (which uses a mismatch penalty of 4 and gap penalty of 6). We decided to use this algorithm for its higher computational speed compared to for example mapping by blast and as such used other parameters for the analyses. While we like the idea of recruitment plots for certain analyses, we believe that this might be more interesting for a detailed study on one of the candidate phyla as it would be difficult to choose a representative view from the amount of scaffolds used within the current study.

23. Lines 558-560: very clear explanation (something rather unusual in this kind of works).

Thanks a lot for this comment, writing detailed methods for this kind of work often gets difficult, therefore, we appreciate this comment.

24. Refs 18, 29, 39: title is capitalized. Please, correct.

This was changed

25. Figure S4: since the discussion about Bacteria and Archaea are separated throughout the manuscript, it would be helpful to have two sets of graphs of RPS3 abundance, one for Archaea and one for Bacteria, or just a different color codes for them in the current graph.

A color-code was added to Figure S4.

26. Table S5 caption: "The order in the table reflects the order of the tree provided in Supplementary Figure 1". This must be a mistake since Suplementary Fig 1 shows "Temperature variability in GB sediments"

Thanks for noticing this, this should indeed refer to Supplementary Fig 2 and was changed accordingly in the caption (now Supplementary Data 6).

Reviewers' Comments:

Reviewer #1:
Remarks to the Author:
My issues have all been addressed.

Reviewer #2:
Remarks to the Author:
I am pleased by how the authors addressed my concerns. The change in title is appropriate and the paper now contains important new discussion to address concerns over low sample number, the strength of environment-diversity linkages, and functional redundancy. While I would still like to see more information about the other five new phyla, I understand the authors' reasoning for structuring the manuscript as they did, and I look forward to future papers that fill in the gaps.

I remain impressed by this work. I believe it makes an important contribution to our knowledge of microbial diversity in the unique Guaymas habitat (and in general). Moreover, it will stand as a model for how to critically analyze this diversity.

Reviewer #3:
Remarks to the Author:
All my points have been addressed satisfactorily (I just saw a typo in line 44). I find this (excelent) manuscript ready for publication.

Detailed point-to-point response to reviewer comments

Reviewer #1 (Remarks to the Author):

My issues have all been addressed.

Reviewer #2 (Remarks to the Author):

I am pleased by how the authors addressed my concerns. The change in title is appropriate and the paper now contains important new discussion to address concerns over low sample number, the strength of environment-diversity linkages, and functional redundancy. While I would still like to see more information about the other five new phyla, I understand the authors' reasoning for structuring the manuscript as they did, and I look forward to future papers that fill in the gaps.

I remain impressed by this work. I believe it makes an important contribution to our knowledge of microbial diversity in the unique Guaymas habitat (and in general). Moreover, it will stand as a model for how to critically analyze this diversity.

Reviewer #3 (Remarks to the Author):

All my points have been addressed satisfactorily (I just saw a typo in line 44). I find this (excelent) manuscript ready for publication.

Thanks for spotting this, the typo in Ln 44 was corrected.
